# Supplementary material for: Intracristal space proteome mapping using super-resolution proximity labeling with isotope-coded probes
Source: Nat Commun. 2025 Aug 20;16:7757. doi: 10.1038/s41467-025-62756-0 (PMC12368266; doi:10.1038/s41467-025-62756-0)
Supplement: Supplementary file 1 — Supplementary Information [file 41467_2025_62756_MOESM1_ESM.pdf]

Supplementary Information

Intracristal space proteome mapping using super-resolution proximity labeling with isotope-coded probes

Myeong-Gyun Kang[1†], Sanghee Shin[2,6,7†], Dong-Gi Jang[2,5], Ohyeon Kwon[3], Song-Yi Lee[1,8], Pratyush Kumar Mishra[1], Minkyo Jung[4], Ji Young Mun[4], Jung-Min Kee[3*], Jong-Seo Kim[2,5*], Hyun-Woo Rhee[1,2*]

[1]Department of Chemistry, Seoul National University, Seoul 08826, Korea

[2]School of Biological Sciences, Seoul National University, Seoul 08826, Korea

[3]Department of Chemistry, Ulsan National Institute of Science and Technology (UNIST), Ulsan 44919, Korea

[4]Neural Circuit Research Group, Korea Brain Research Institute, Daegu 41062, Republic of Korea

[5]Center for RNA Research, Institute for Basic Science, Seoul 08826, Korea

[6]The Research Institute of Basic Science, Seoul National University, Seoul 08826, Korea

[7]Department of Cancer Biology, Dana-Farber Cancer Institute, Boston, MA, USA

[8]Department of New Biology, Daegu Gyeongbuk Institute of Science & Technology (DGIST), Daegu 42988, Korea

[9]New Biology Research Center, Daegu Gyeongbuk Institute of Science & Technology (DGIST), Daegu 42988, Korea

[†]These authors equally contributed to this work.

*Corresponding authors. Email: rheehw@snu.ac.kr (H.-W.R.); jongseokim@snu.ac.kr (J.-S.K.); jmkee@unist.ac.kr (J.-M.K.)

Supplementary Figures

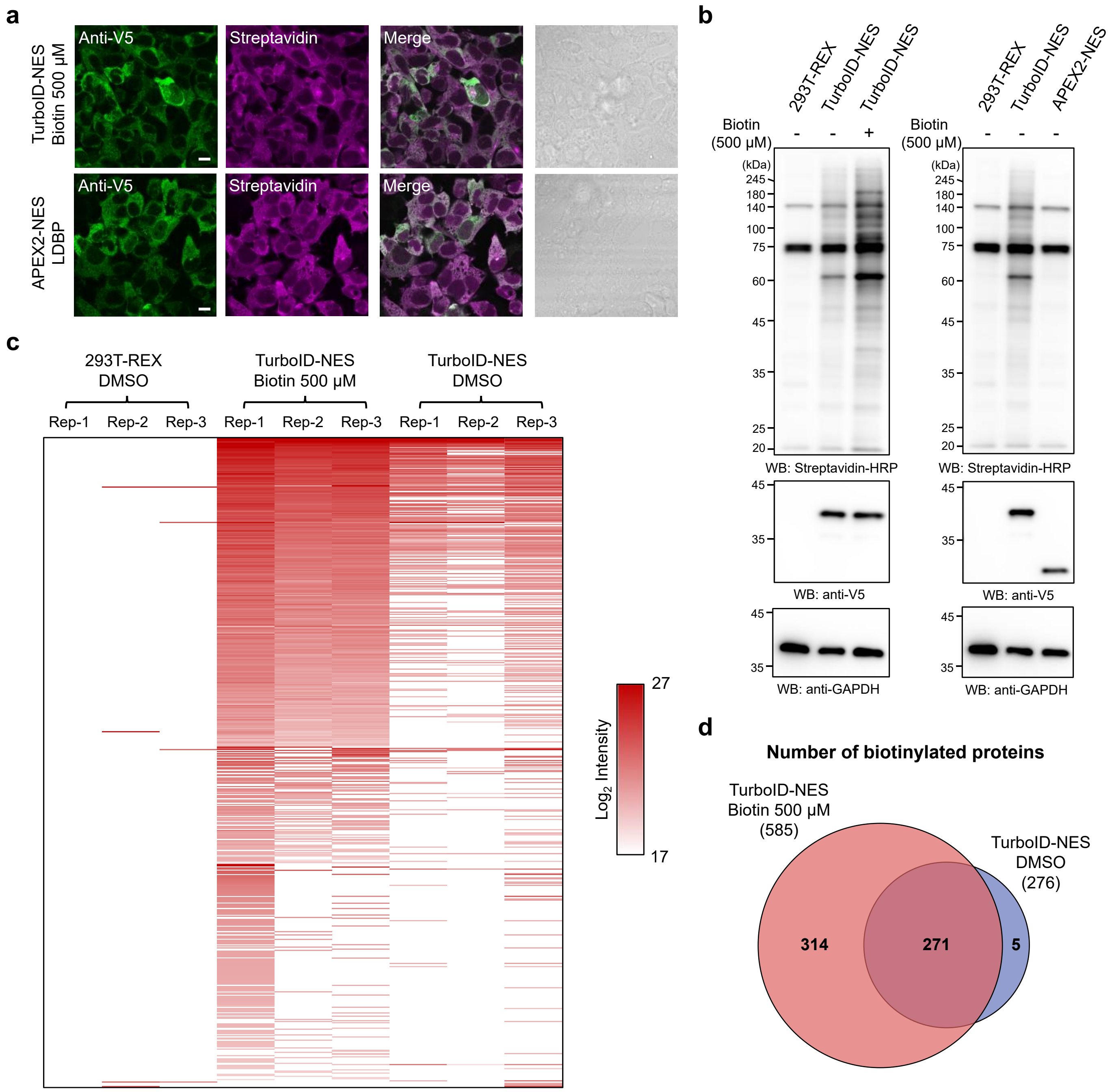

Supplementary Figure 1. TurboID exhibits high reactivity with endogenous biotin levels. (**a**) Imaging analysis of TurboID-NES and APEX2-NES. Proximity labeling enzymes localize in the cytosol. (**b**) Western blotting of TurboID-NES and APEX2-NES shows the biotinylation level after treatment with DMSO and 500 µM biotin. TurboID and APEX2 levels were validated using anti-V5 antibody. GAPDH was used for loading control. (**c**) LC-MS/MS analysis of biotin-modified peptides after peptide-level enrichment. Heat map of identified proteins biotinylated by TurboID-NES based on their MS1 intensity (n = 3 biological replicates). (**d**) Venn diagram comparing the number of proteins biotinylated by TurboID-NES between DMSO-treated and excess biotin-treated samples.

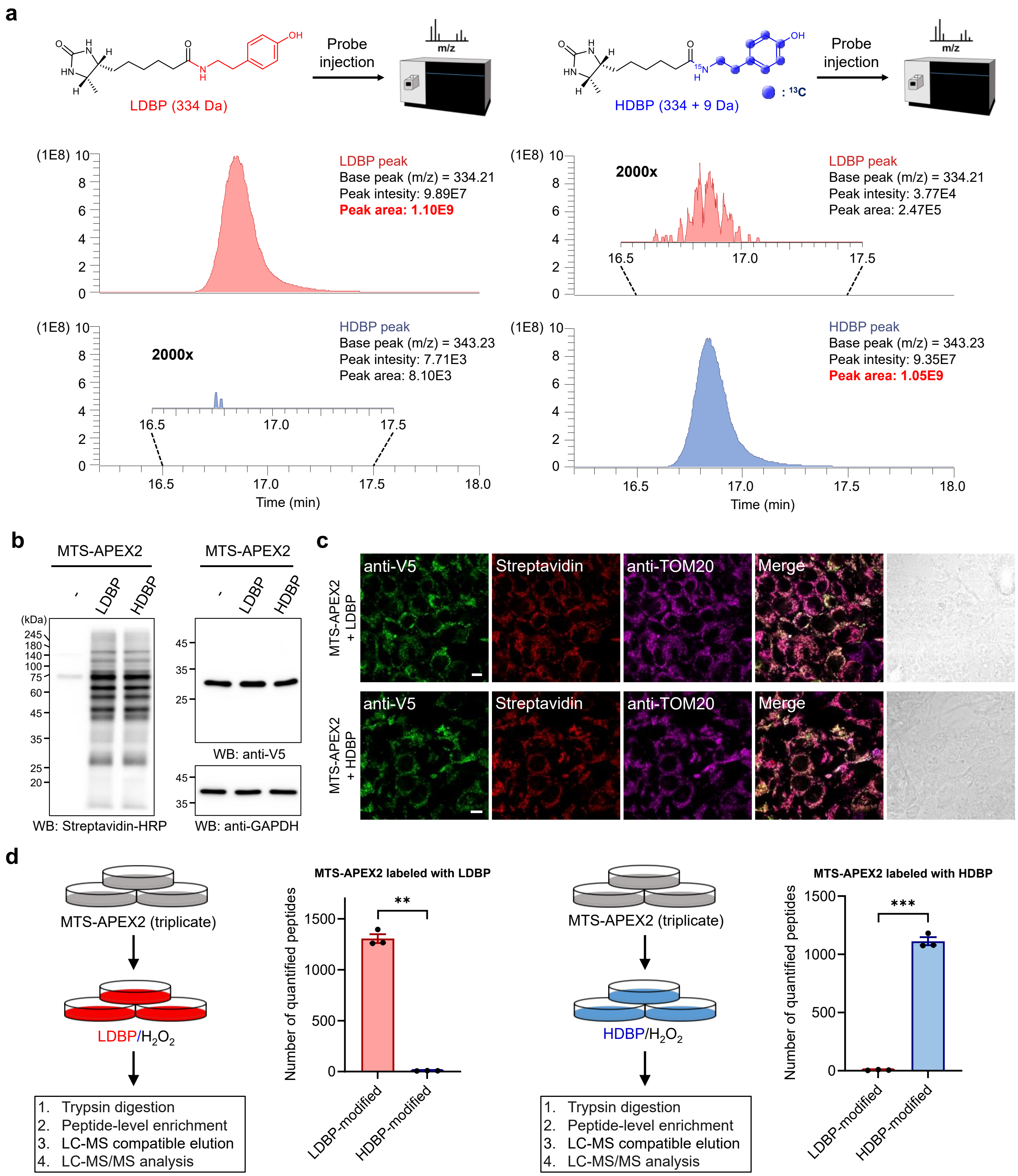

Supplementary Figure 2. Isotopic purity of the synthesized LDBP and HDBP probes. (**a**) LC-MS analysis of LDBP and HDBP probes after direct injection. Peak intensity and area were used to calculate isotopic purity. (**b**) Western blotting shows biotinylation level of proteins labeled by MTS-APEX2 using LDBP and HDBP. (**c**) Imaging analysis of MTS-APEX2 shows their localization and biotinylation pattern. Scale bars represent 10 μm. (**d**) Bar graph of the numbers of LDBP- and HDBP-modified peptides identified in either LDBP- or HDBP-treated samples after peptide-level enrichment (n = 3 biological replicates). The *p*-values were calculated using an unpaired two-tailed Welch's *t*-test. Statistical significance was indicated as following: $**p < 0.01$ and $***p < 0.001$. Mean values are shown with error bars representing the standard error of the mean, and each dot indicates the individual data points.

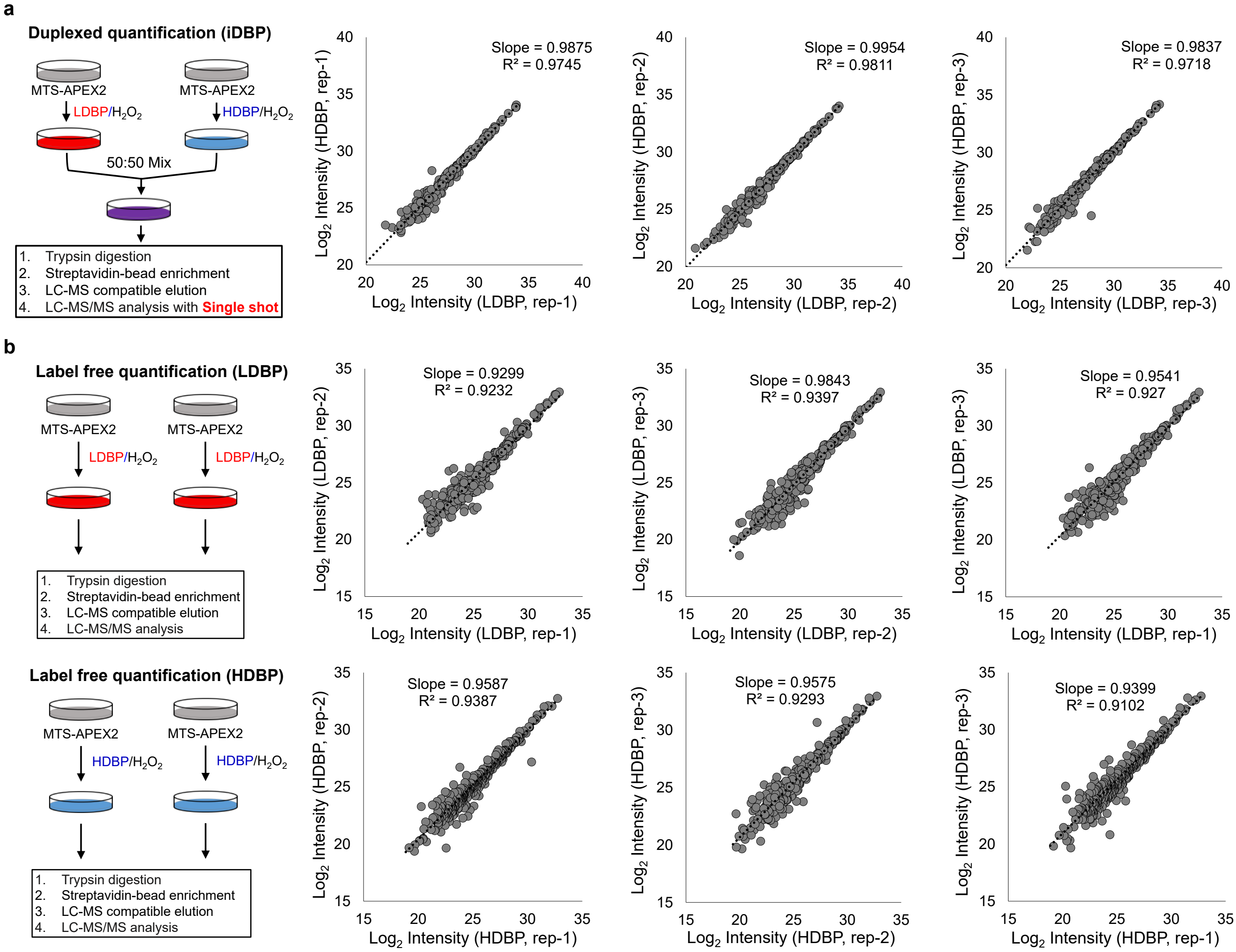

Supplementary Figure 3. Accuracy and reproducibility of duplexed quantification using ICAX with isotope-coded DBP probes (iDBP) vs. label free quantification (LFQ). (**a**) ICAX was conducted by single analysis with a mixture of LDBP- and HDBP-modified peptides by MTS-APEX2 after enrichment. Scatter plot is based on MS1 intensity. R-squared (R^2) values and slopes of trendlines are displayed for validation of accuracy and reproducibility across biological triplicate experiments. (**b**) LFQ analysis was conducted by separately injecting either LDBP- or HDBP-modified peptides labeled by MTS-APEX2. The scatter plot displays R^2 values and trendline slopes obtained from biological triplicate experiments.

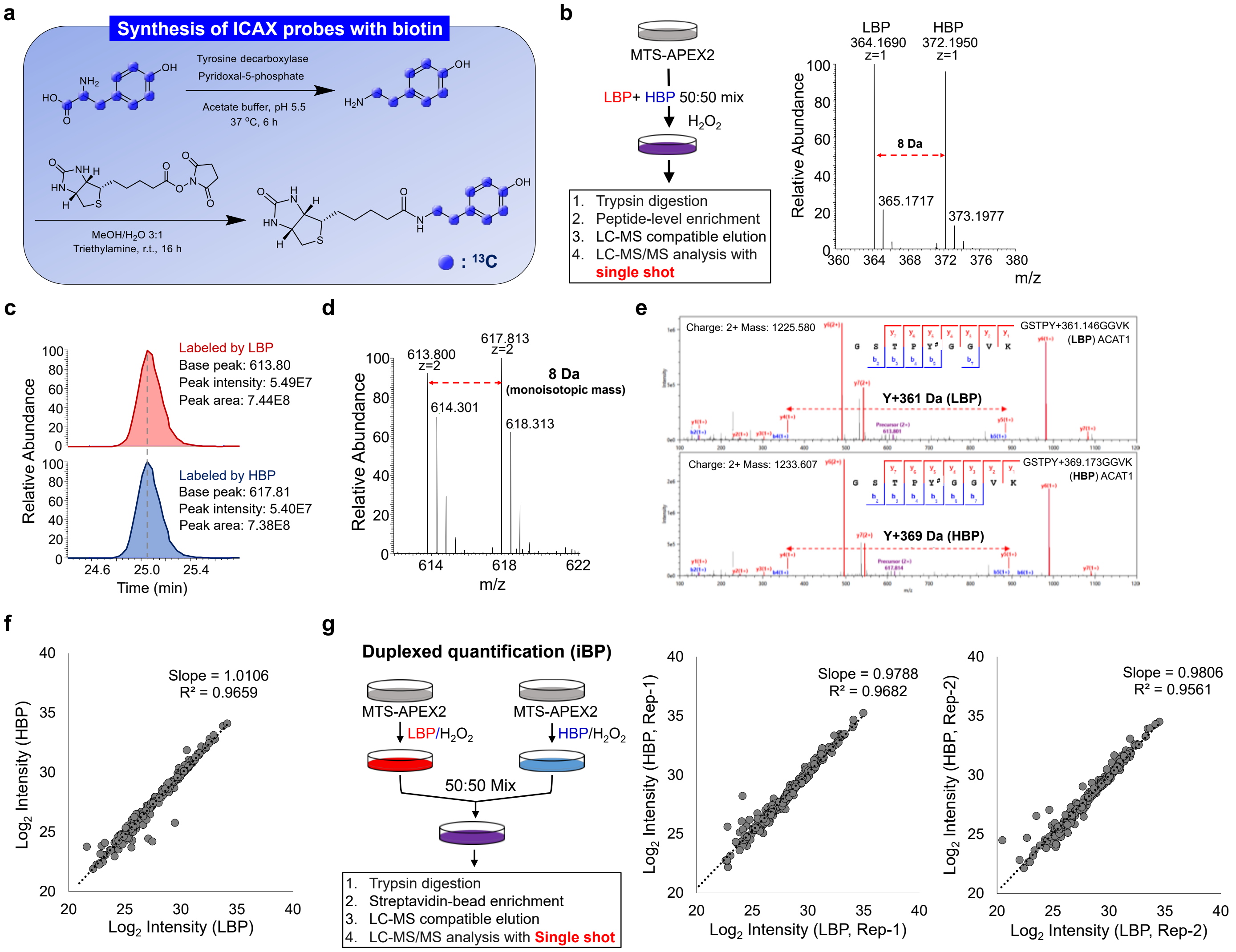

Supplementary Figure 4. Duplexed super-resolution proximity labeling analysis of LBP- and HBP-labeled proteins. (**a**) Chemical structure of heavy isotope-coded ascorbate peroxidase (APEX) probes, heavy biotin-phenol (HBP), used in this study. Isotope-coded biotin-phenol probes (iBP) were synthesized from tyrosine and conjugated with a biotin moiety. (**b**) Equal-activity test of biotin-conjugated APEX probes (light biotin-phenol [LBP] and HBP, 50:50 ratio mixture) on cells expressing mitochondrial targeting sequence (MTS)-APEX2. The right panel shows a high-resolution mass spectrum of protonated LBP and HBP. (**c**) Representative extracted ion chromatograms of LBP- and HBP-labeled peptides (GSTPY#GGVK of the ACAT1 protein) detected by mass spectrometry (MS). The labeled peptide pairs were exactly co-eluted with very similar peak intensities and areas. (**d**) MS1 intensities of the peptide pair labeled with LBP and HBP are shown in the doublet MS spectrum (**e**) Annotated MS/MS spectra of the labeled peptide pair in (**c**). (**f**) MS1 intensity plot of both LBP and HBP proteins, labeled with MTS-APEX2 using a 50:50 probe mixture (n = 2 biological replicates). (**g**) Scatter plot based on MS1 intensity shows R-squared (R^2) values and trendline slopes of biological duplicate ICAX experiments.

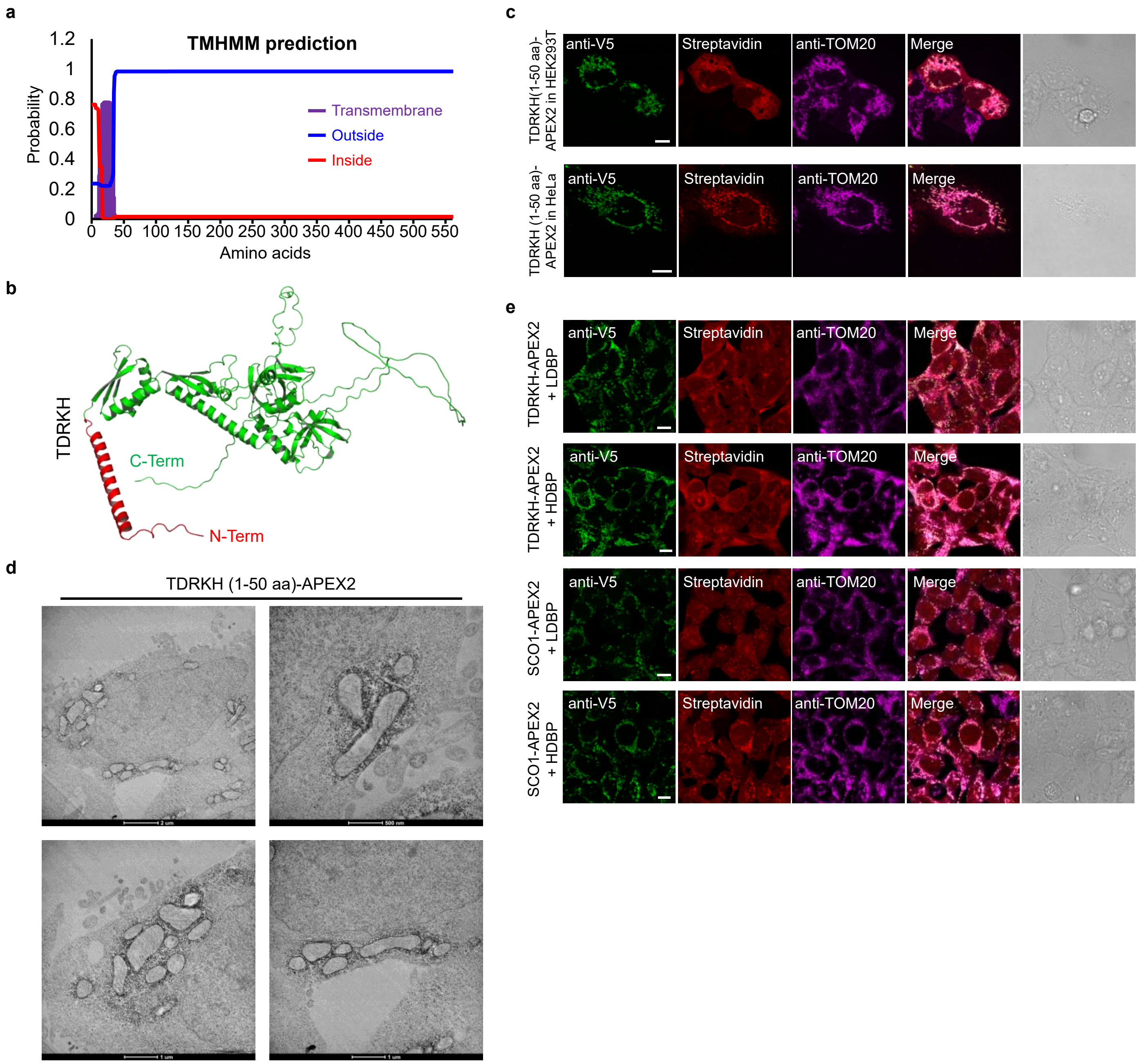

Supplementary Figure 5. Preparation of ascorbate peroxidase (APEX) constructs targeting TDRKH and SCO1 for the identification of primary intracristal space proteome. (**a**) TDRKH transmembrane domain (17–34 aa) predicted using the transmembrane helix hidden Markov model. (**b**) TDRKH (1–50 aa) is indicated in red in the AlphaFold-predicted TDRKH structure (identifier: AF-Q9Y2W6-F1). (**c**) Confocal microscopy images of TDRKH (1–50 aa)-APEX2 in Flp-In T-Rex 293 (upper panel) and HeLa cells (lower panel). (**d**) Electron microscopic images obtained after diaminobenzidine staining for TDRKH (1–50 aa)-APEX2. (**e**) Confocal microscopy images of TDRKH (1–561 aa)-APEX2 and SCO1-APEX2 stable cell lines. Biotinylated proteins were detected using streptavidin-AF568 staining. The expression of each construct was visualized using an anti-V5 antibody. The scale bars in the images represent 10 μm.

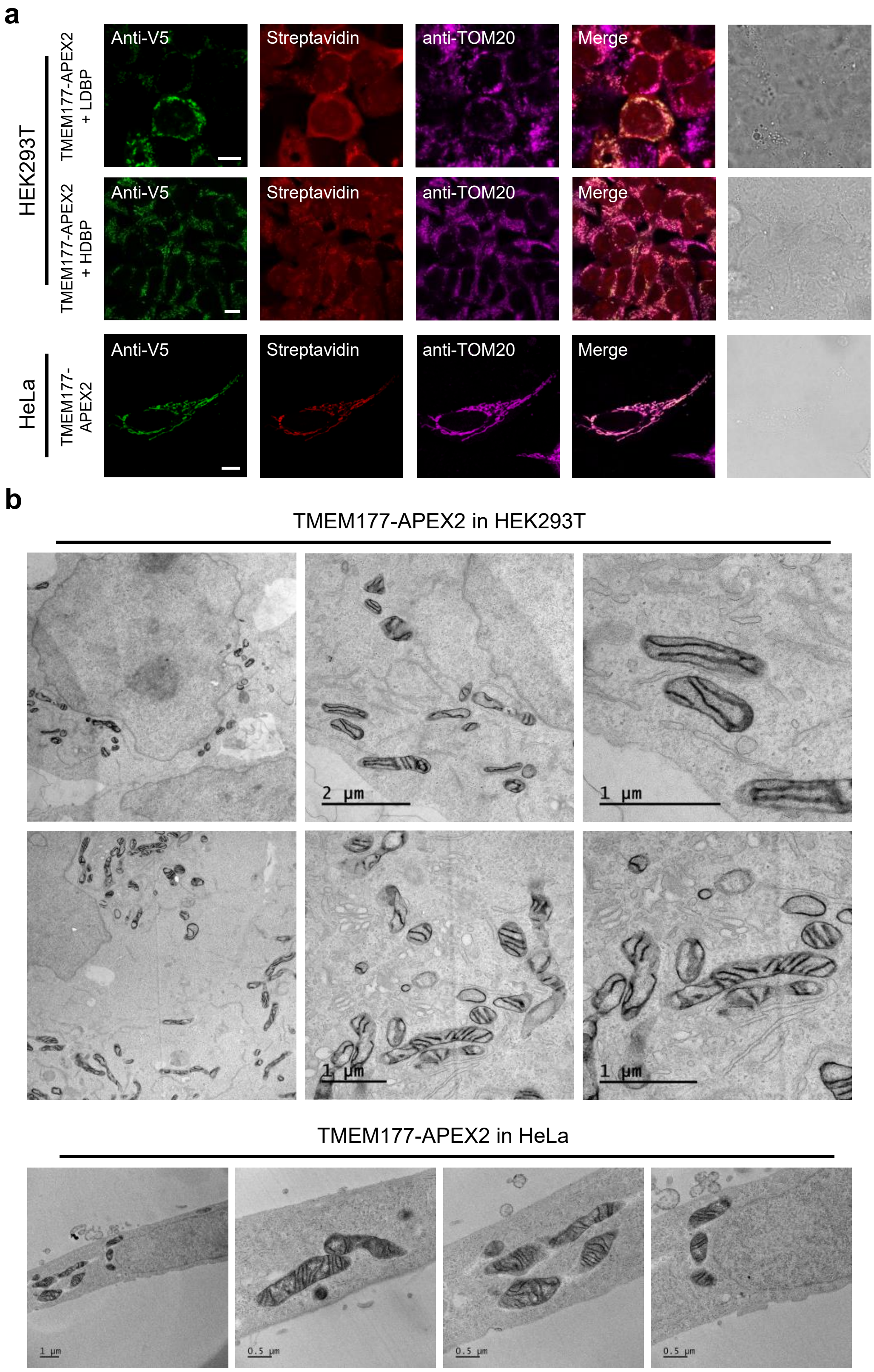

Supplementary Figure 6. TMEM177-APEX2 localization and biotinylation activity. (**a**) TMEM177-APEX2 images were captured using confocal microscopy after light desthiobiotin-phenol (LDBP) and heavy desthiobiotin-phenol labeling in Flp-In T-Rex 293 cells. Biotinylated proteins were stained with streptavidin-AF568, and APEX2 expression was confirmed using an anti-V5 antibody (green). The mitochondrial patterns were visualized using an anti-TOMM20 antibody (magenta). For TMEM177-APEX2 imaging in HeLa cells, biotinylation was performed using LDBP. (**b**) Additional electron microscopic images of TMEM177-APEX2 in Flp-In T-Rex 293 and HeLa cells (**Fig. 2g**).

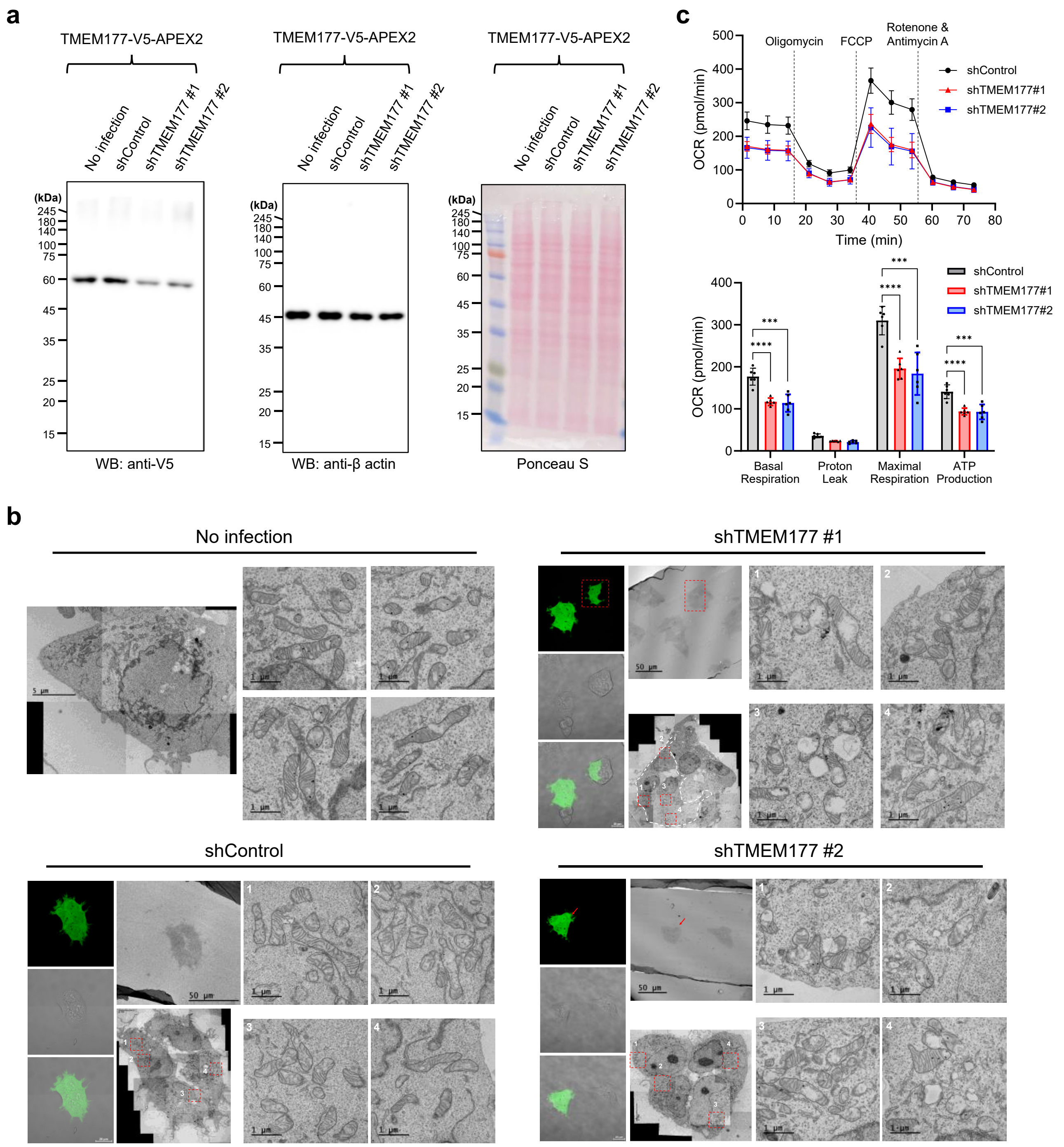

Supplementary Figure 7. Characterization of mitochondrial defects in the TMEM177-deficient cell line. (**a**) Western blot analysis of TMEM177-knockdown (shTMEM177 #1 and shTMEM177 #2) Flp-In T-Rex 293 cells. TMEM177-V5-APEX2 levels were determined using an anti-V5 antibody. Anti-β-actin antibody and ponceau S staining were used as the loading control for western blotting. (**b**) Correlative light and electron microscopic images of wild-type (non-infected), shControl-, and shTMEM177-infected Flp-In T-Rex 293 cells. The mitochondrial morphologies are highlighted in these images. (**c**) Oxygen consumption rate (OCR) of control and TMEM177-deficient Flp-In T-Rex 293 cells over time at the basal level and following the addition of oligomycin (1.5 μM), FCCP (0.5 μM), or rotenone (0.5 μM). Quantitative analyses of basal respiration, proton leakage, maximal respiration, and ATP production among the cells (bar graph; n = 6 biological replicates per group). Mean values are shown with error bars representing the standard deviation. Dots in the bar graph indicate individual data points. Statistical significance was calculated using an unpaired two-tailed Student's *t*-test (***p < 0.001, ****p < 0.0001).

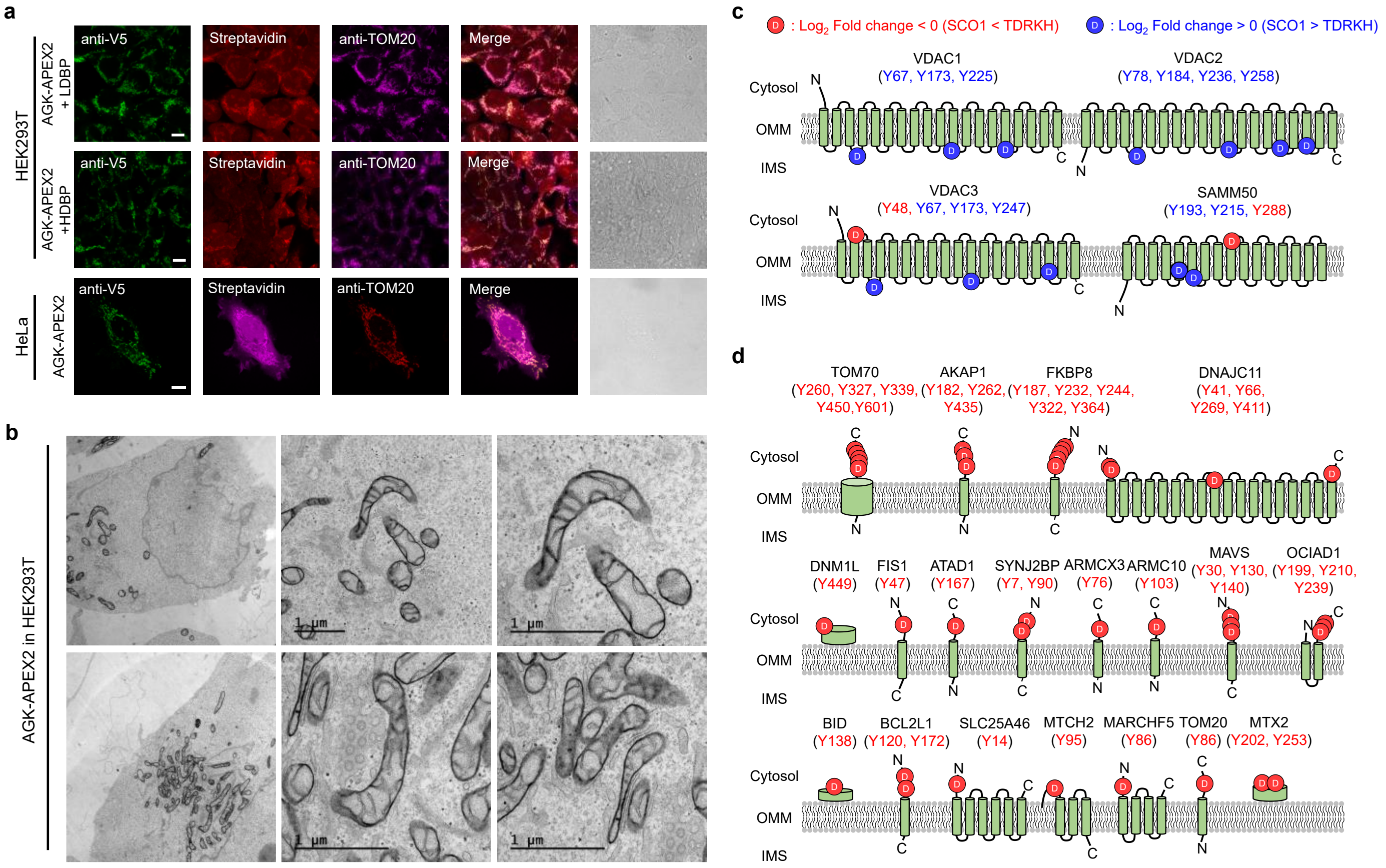

Supplementary Figure 8. Characterization of the AGK-APEX2 construct and proposed membrane topology of outer-mitochondrial-membrane proteins based on the FC_SCO1/TDRKH of ICAX-labeled proteins. (**a**) Confocal microscopy images of AGK-APEX2 and its biotinylated proteins in Flp-In T-Rex 293 and HeLa cells. To determine whether light desthiobiotin-phenol (LDBP) and heavy desthiobiotin-phenol labeling patterns were identical, biotinylation was performed using both the probes in Flp-In T-Rex 293 cells. The biotinylated proteins were stained with streptavidin-AF568 or AF647. (**b**) Additional transmission electron microscopic images of AGK-APEX2 after diaminobenzidine staining (related to **Figure 2g**). Topology of mitochondrial outer membrane proteins with our identified modification site information in groups II (**c**) and III (**d**). DBP-labeled sites (D) colored red indicates negative fold-change (FC) values; that is, they were labeled to a greater extent by TDRKH-APEX2, whereas DBP colored blue indicates positive FC values; that is, they were labeled to a greater extent by SCO1-APEX2. The scale bars in the confocal microscopy images represent 10 μm. Detailed information is provided in **Supplementary Data 6**.

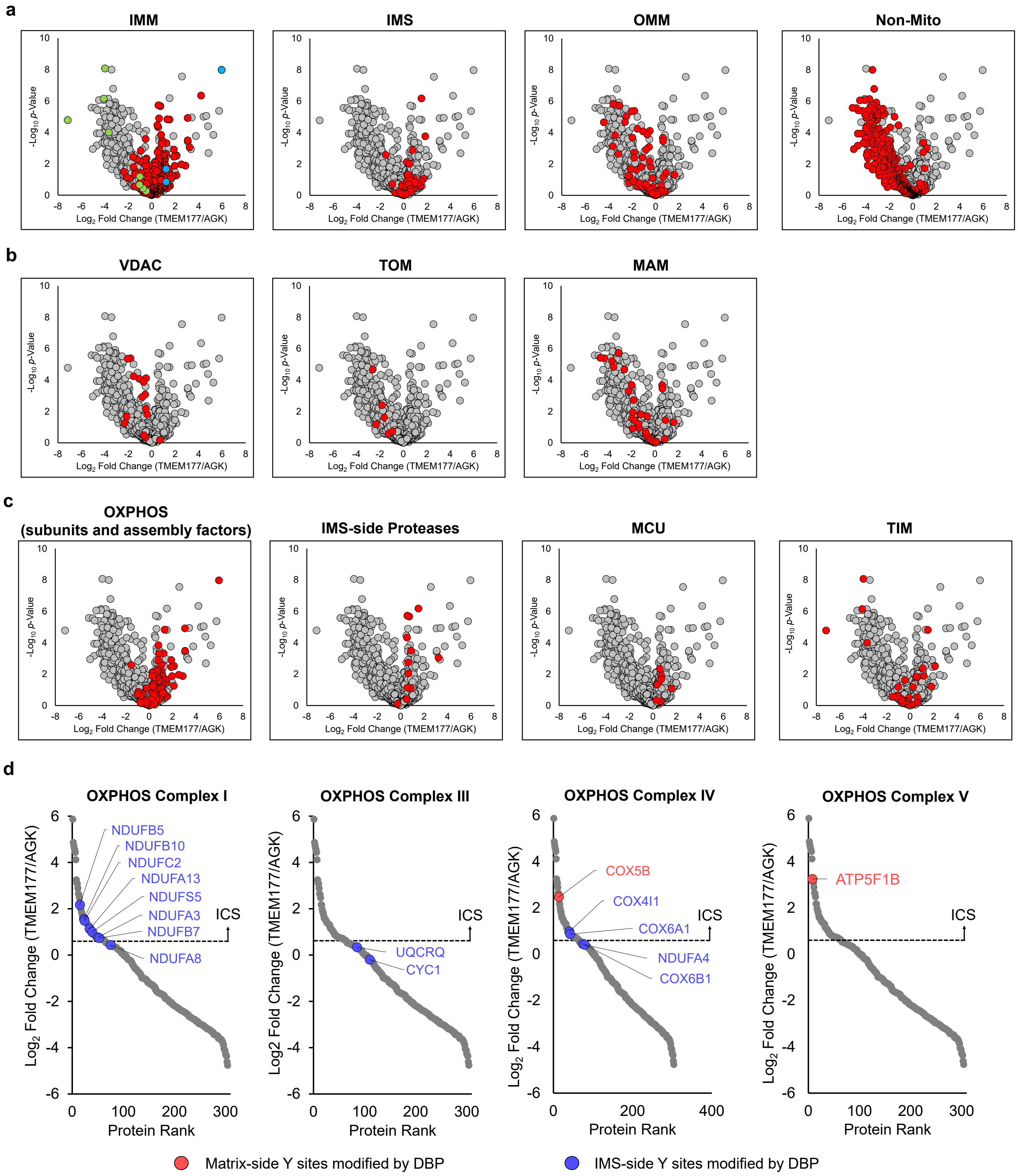

Supplementary Figure 9. Validation of the identified ICS proteins using isotope-coded phenol probes for ascorbate peroxidase (APEX) labeling on TMEM177- and AGK-APEX2 stable cell lines. (**a–c**) Volcano plot of proteins labeled with isotope-coded phenol probes using TMEM177-APEX2 or AGK-APEX2. In the volcano plot for the inner mitochondrial membrane, the blue dots indicate TMEM177, and the green dots indicate AGK (n = 4 biological replicates). (**d**) The scatter plot shows the distribution of subunits in OXPHOS complexes I, III, IV, and V. Protein modification sites exposed to the mitochondrial matrix and IMS are marked in red and blue, respectively (n = 4 biological replicates).

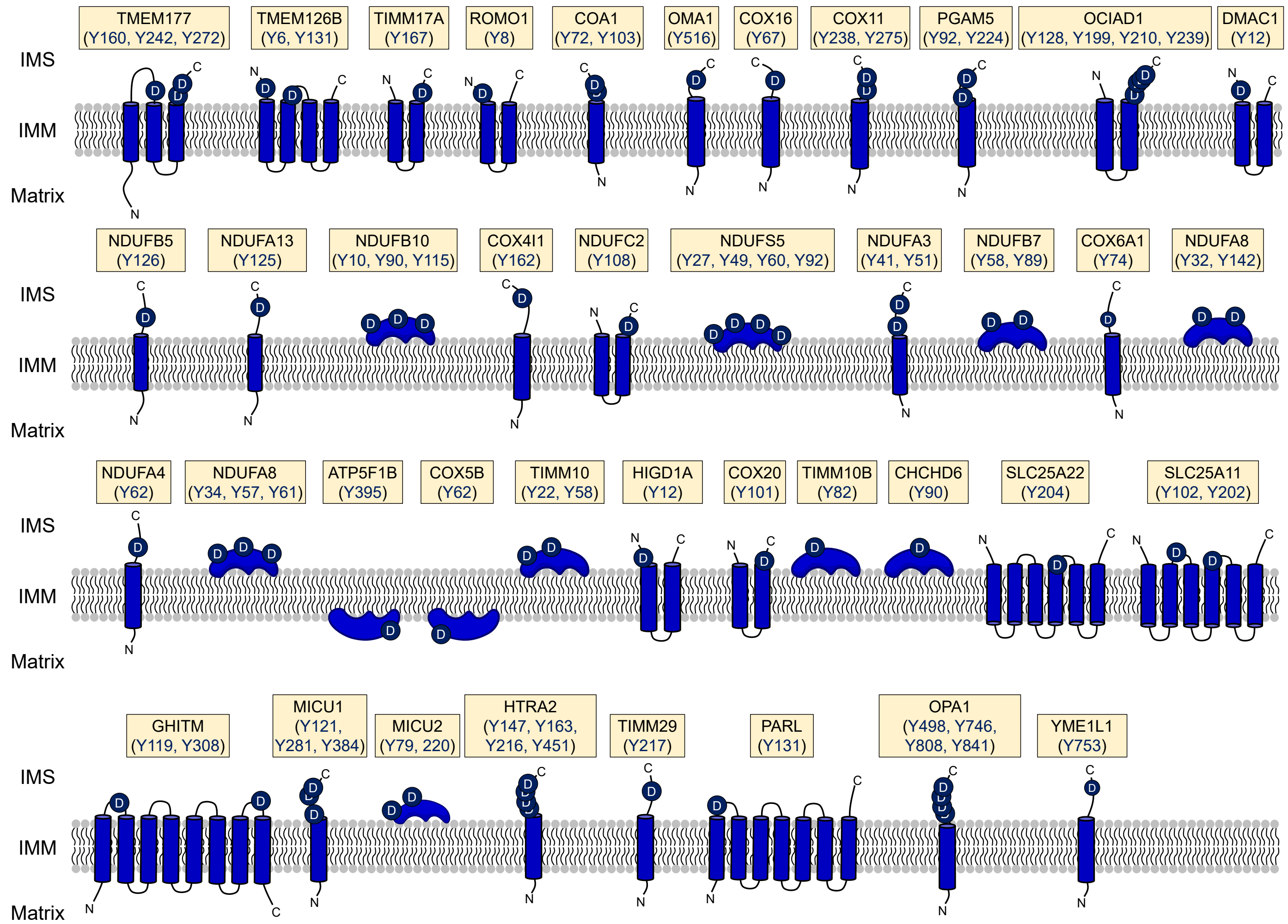

Supplementary Figure 10. Membrane topology of mitochondrial inner membrane-oriented proteins observed in the ICS group.

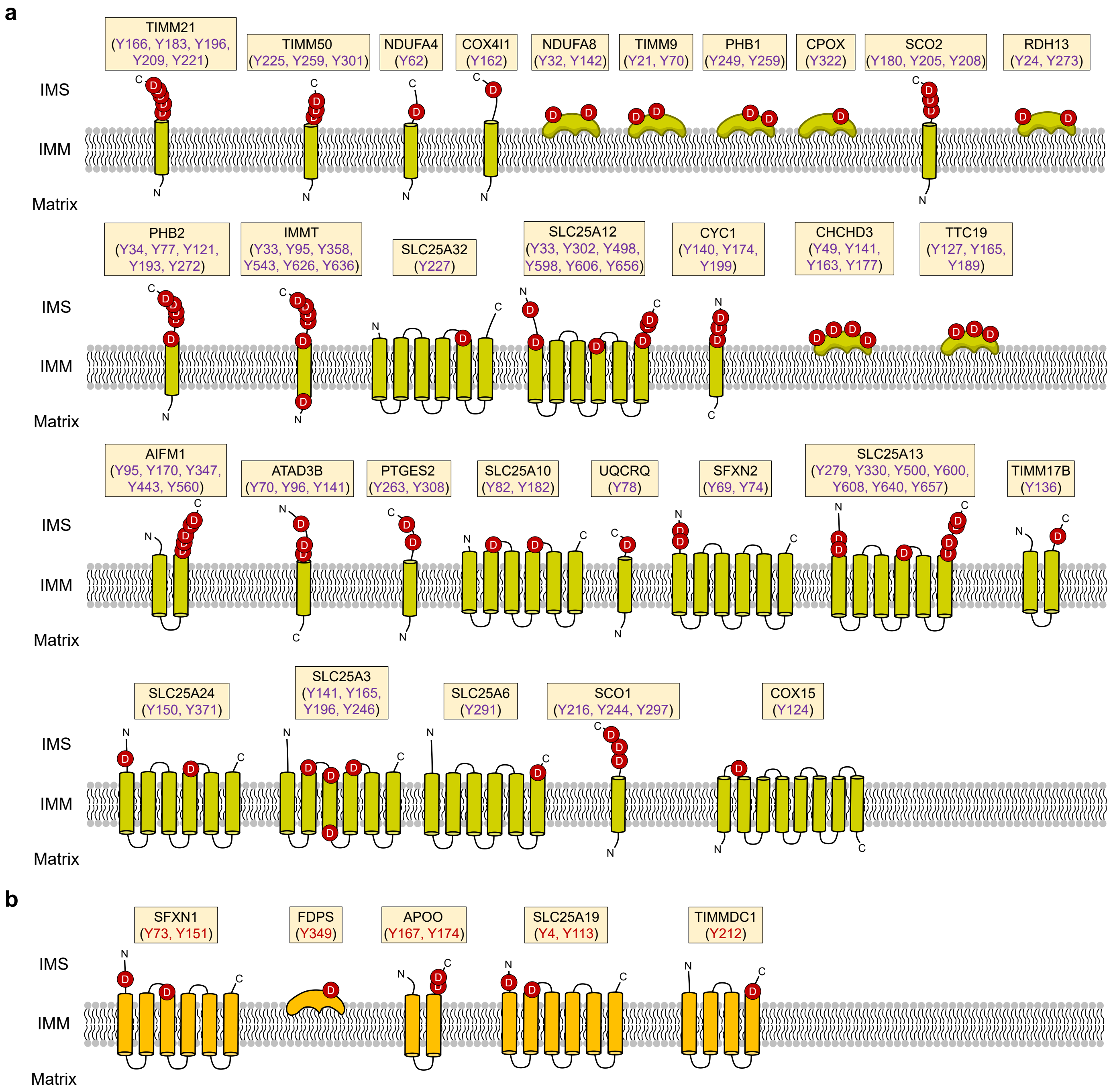

Supplementary Figure 11. (**a, b**) Membrane topology of mitochondrial inner membrane-oriented proteins observed in the OCS group (**a**) and OMM/cytosol group (**b**).

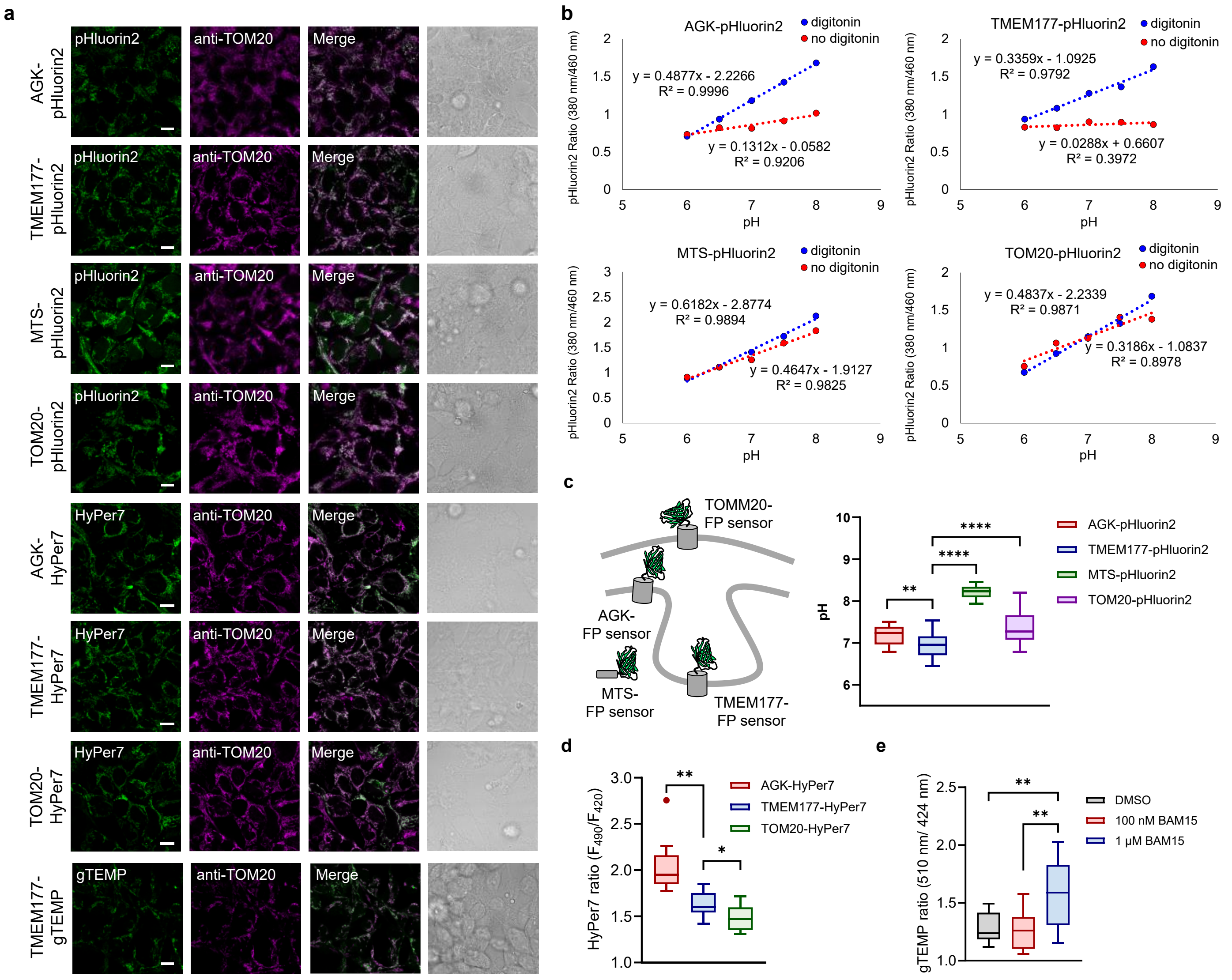

Supplementary Figure 12. Assessment of intracristal and outer intracristal subdomain microenvironments and their correlations with the structural properties of their corresponding proteomes. (**a**) Confocal microscopy images of various fluorescent-protein, sensor-conjugated TMEM177 and AGK constructs. All scale bars represent 10 µm. (**b**) Titration curves for each pHluorin2 construct, with and without digitonin treatment (n = 6 biological replicates per group). (**c**) Schematic representation of the fluorescent proteins targeting each submitochondrial compartment for microenvironment assessment. The pH was measured with pHluorin2, which targets the outer intracristal space (OCS) (AGK), intracristal space (ICS) (TMEM177), mitochondrial targeting sequence (MTS), and cytoplasmic side of mitochondria (TOM20) (n = 24 biological replicates per group). (**d**) The redox state was measured with HyPer7 targeting the OCS, ICS, and outer mitochondrial membrane facing the cytoplasm (TOM20) (n = 10 biological replicates per group). (**e**) The relative ICS temperature was measured using TMEM177-gTEMP following treatment with 0.1% DMSO or BAM15 (n = 11 biological replicates per group). Tukey style box-and-whisker plots were used in (**c-e**), and dot represent the outlier in (**d**). The *p*-values in (**c-e**) were calculated using an unpaired two-tailed Welch's *t*-test. Statistical significance was indicated as following: $*p < 0.05$, $**p < 0.01$, $****p < 0.0001$.

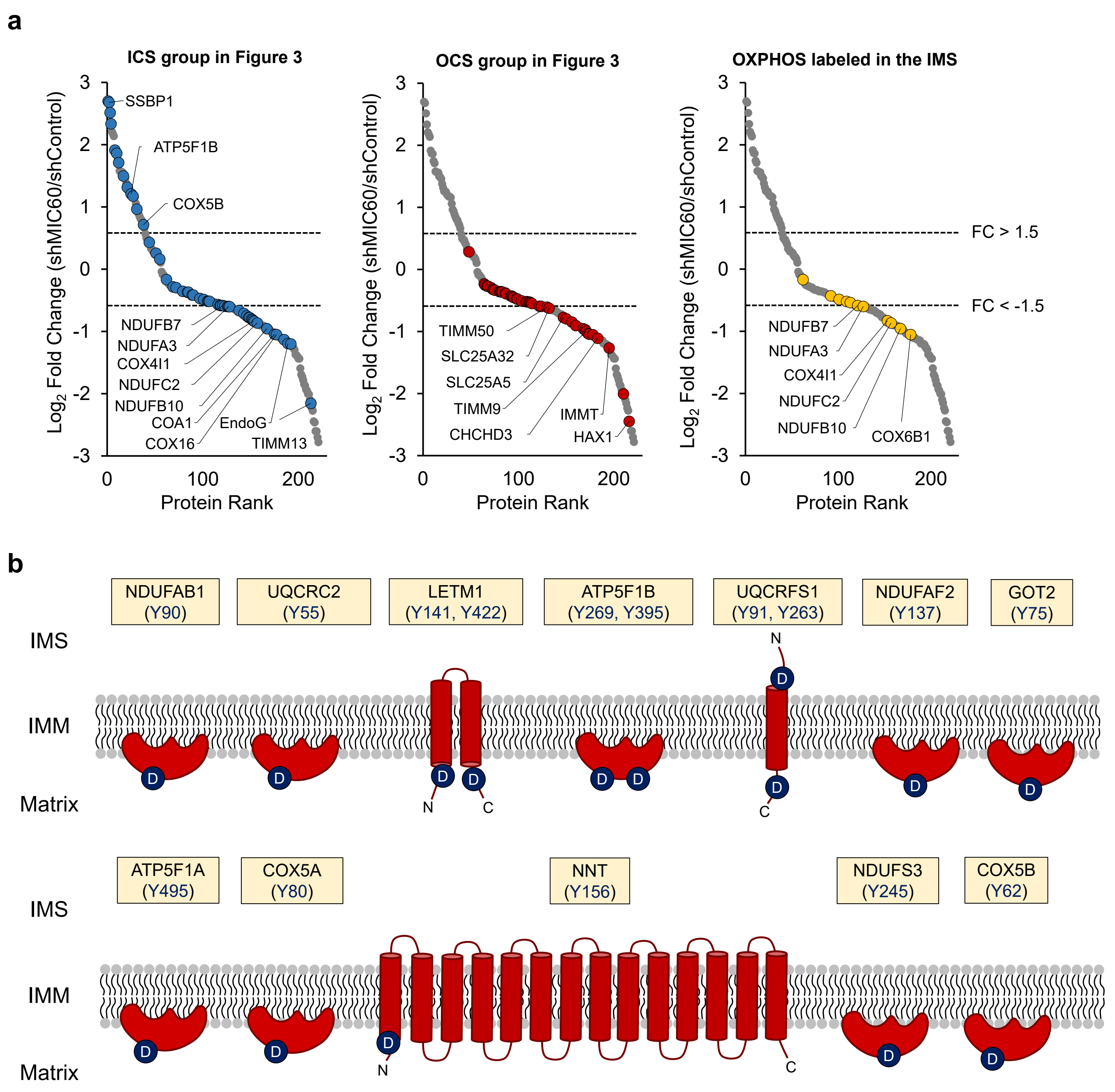

Supplementary Figure 13. (**a**) Scatter plot showing the list of ICS and OCS proteome identified in TMEM177-, and AGK-APEX2 dataset. OXPHOS subunits with IMS-side DBP modification are shown in the right panel. (**b**) Membrane topology of IMM proteins showing matrix-side modification by diffused DBP from ICS under MICOS complex inhibition.

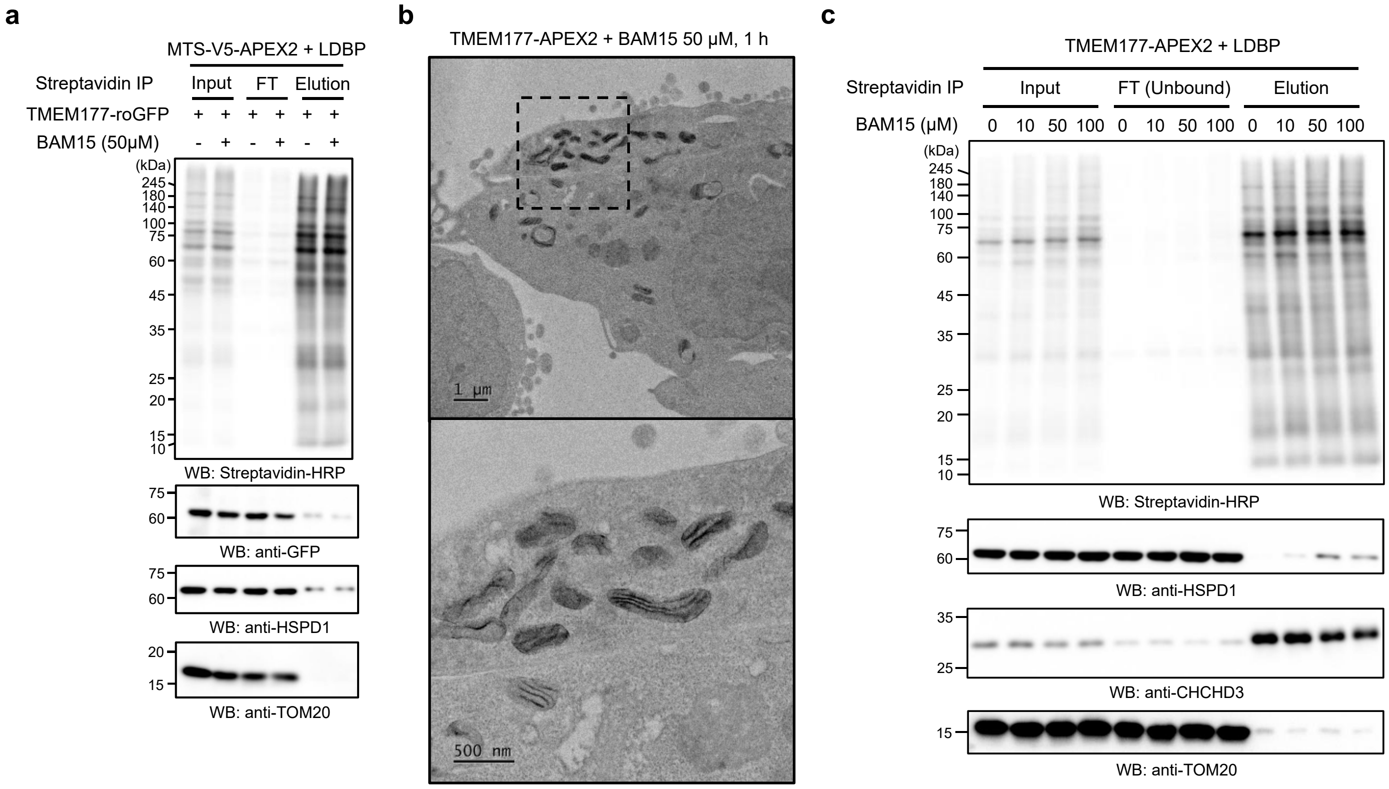

Supplementary Figure 14. Characterization of the topological domain of TMEM177-APEX2 under BAM15 treatment. (**a**) Proximity biotinylation of MTS-APEX2 for TMEM177-conjugated fluorescent protein (roGFP) with or without BAM15 treatment. Biotinylated TMEM177-roGFP was detected via Western blotting using an anti-GFP antibody after enrichment with streptavidin-conjugated beads. The labeling of mitochondrial matrix proteins by MTS-APEX2 was confirmed through immunoblotting with anti-HSPD1 (mitochondrial matrix) and anti-TOM20 (outer mitochondrial membrane) antibodies. The proximal biotinylation level of TMEM177-roGFP labeled with MTS-APEX2 remained unchanged following BAM15 treatment. (**b**) Electron microscopic images of TMEM177-APEX2 after diaminobenzidine staining under mitochondrial uncoupling. (**c**) Biotinylated proteins, including HSPD1, labeled with TMEM177-APEX2 were detected following treatment with various concentrations of BAM15.

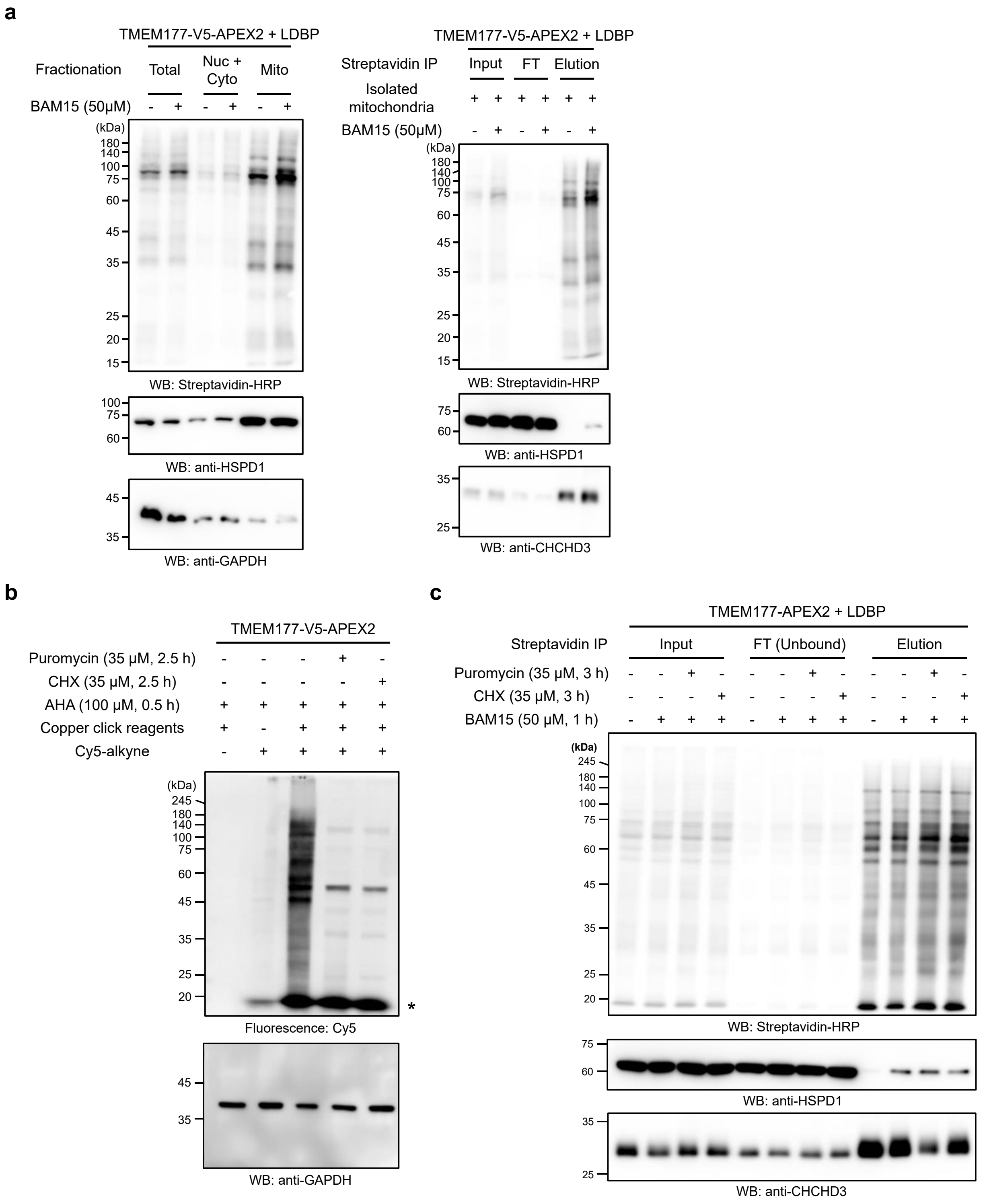

Supplementary Figure 15. Determination of the localization of biotinylated proteins labeled by TMEM177-APEX2 under BAM15 treatment. (**a**) Detection of biotinylated proteins labeled by TMEM177-APEX2 in isolated mitochondria (left panel). Biotinylated proteins in isolated mitochondria were enriched using streptavidin-conjugated beads following DMSO or BAM15 treatment (right panel). (**b**) Measurement of the protein synthesis level using L-azidohomoalanine (AHA) after puromycin and cycloheximide (CHX) treatment. AHA-incorporated proteins were labeled with Cy5-alkyne by click reaction for detection. Asterisk indicates the unreacted Cy5-alkyne. (**c**) Enrichment of biotinylated proteins labeled by TMEM177-APEX2 using streptavidin-conjugated beads under the mitochondrial uncoupling conditions. Cells were treated with puromycin and CHX for 2 h followed by addition of BAM15 and LDBP for 1 h.

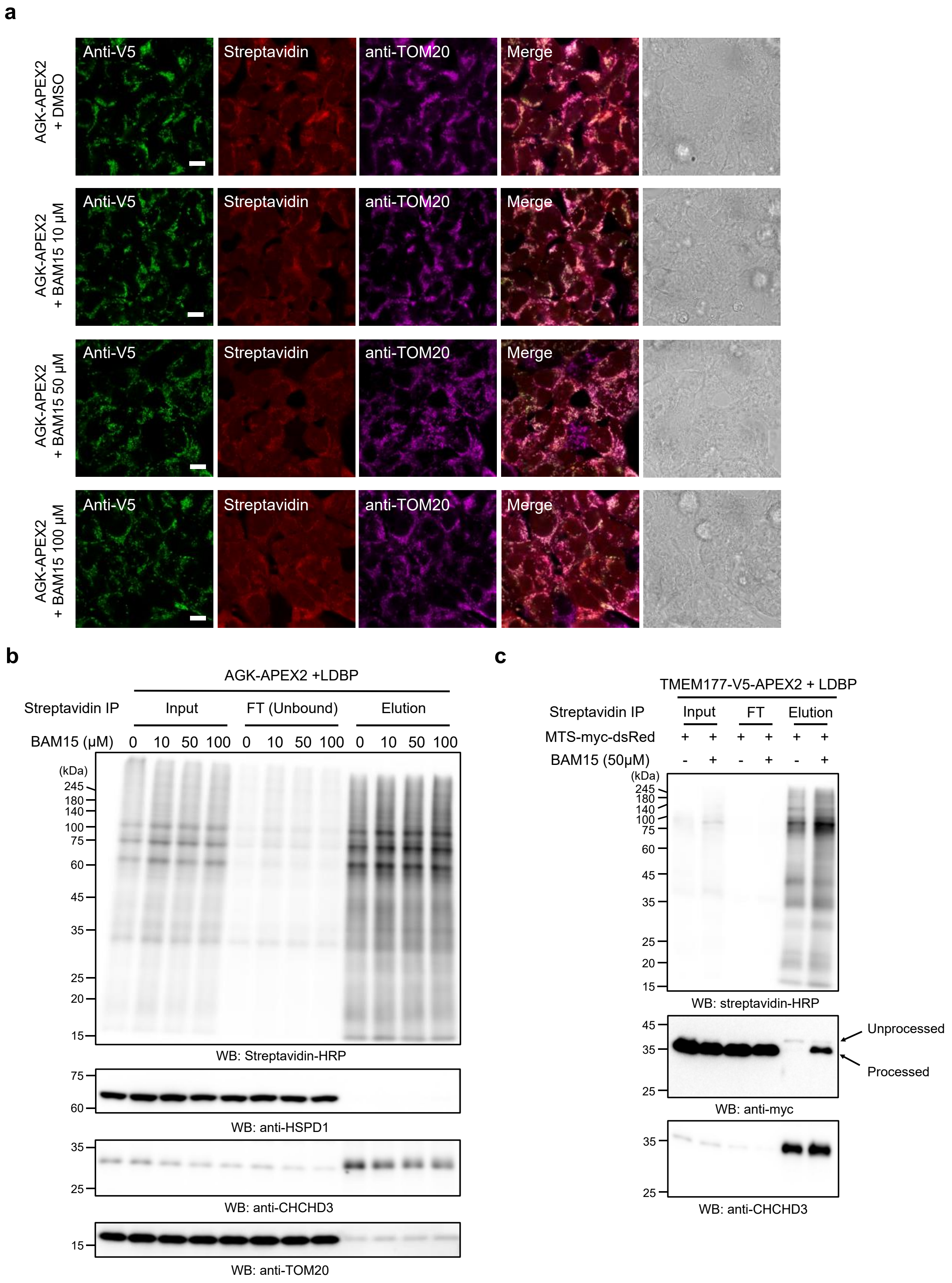

Supplementary Figure 16. TMEM177-APEX2-specific labeling of mitochondrial matrix proteins. (**a, b**) Confocal microscopy images (**a**) and Western blot analysis (**b**) exhibit biotinylated proteins labeled by AGK-APEX2 following treatment with various concentrations of BAM15 for 1 h. The scale bars in the images represent 10 μm. (**c**) Western blot analysis shows that the processed form of MTS-dsRed, cleaved by mitochondrial processing peptidase that, was strongly biotinylated by TMEM177-APEX2 under mitochondrial uncoupling conditions.

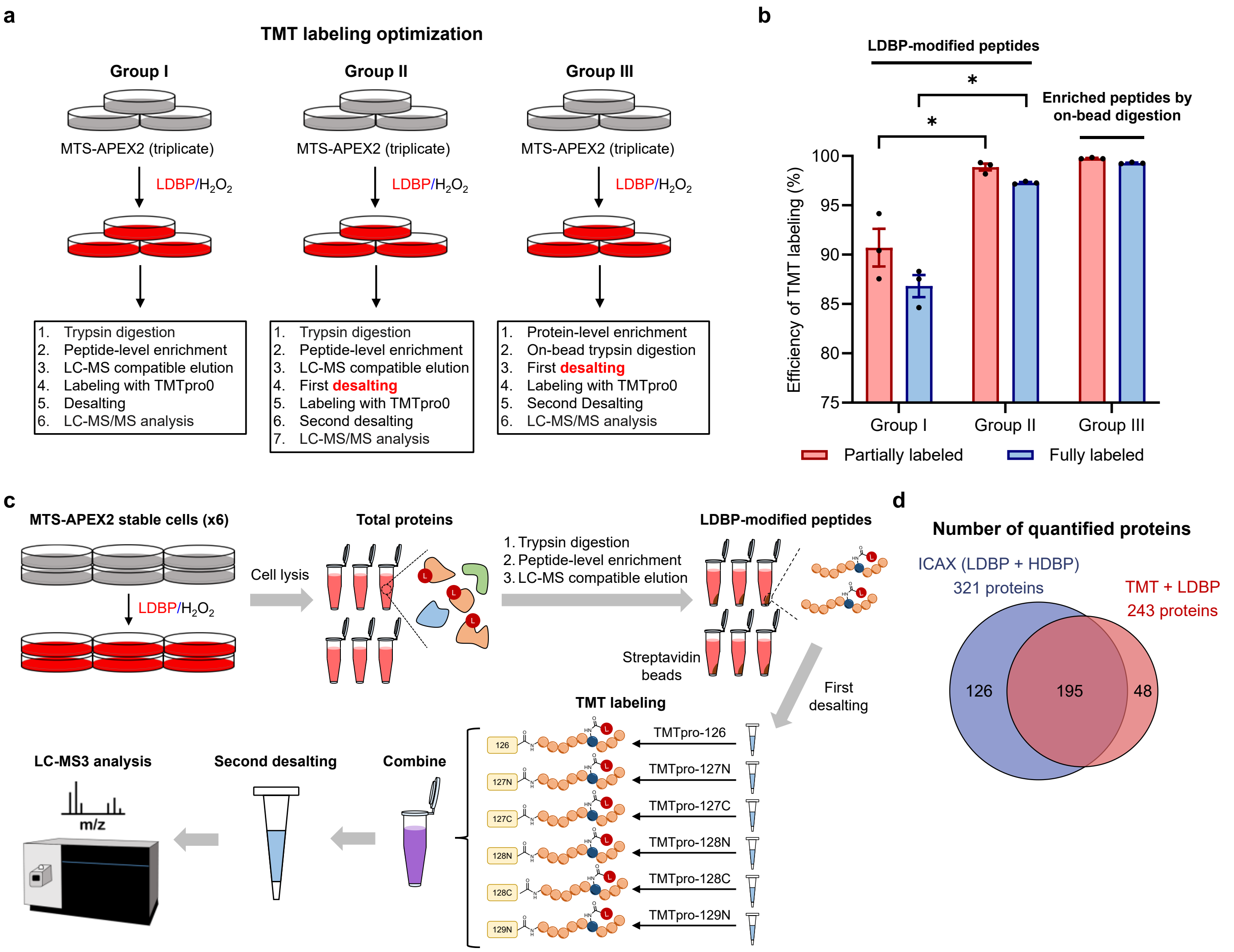

Supplementary Figure 17. Comparison between ICAX and isobaric quantification using TMT labeling. (**a**) Schematic representation of the LC-MS/MS sample procedure for TMT labeling after peptide-level and conventional protein-level enrichment. (**b**) Bar graphs showing the TMT labeling efficiency in each group. Partially labeled peptides indicate that TMT was labeled at least on the N-terminus or lysine residues. In this triplicate experiment, TMTpro0 was used for labeling. The *p*-values for triplicate experiments were calculated using an unpaired two-tailed Welch's *t*-test. Statistical significance was indicated as following: $*p < 0.05$. Mean values are shown with error bars representing the standard error of the mean. Dots indicate individual data points. (**c**) Schematic representation of the LC-MS3 analysis with TMT-labeled samples for LDBP-modified peptides labeled by MTS-APEX2. Peptide desalting was performed for TMT labeling and removal of residual TMT after reaction. (**d**) Venn diagram showing the number of quantified proteins using TMT labeling compared with the ICAX results.

a

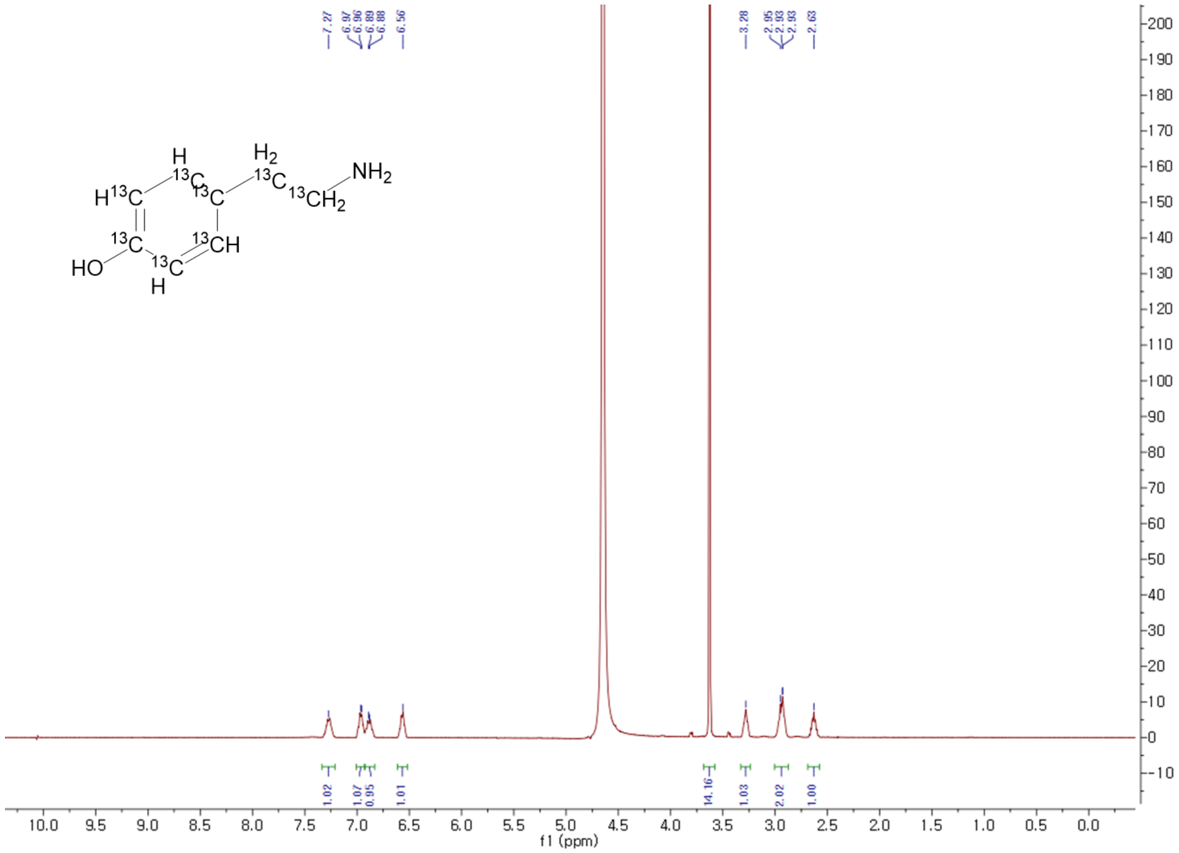

b

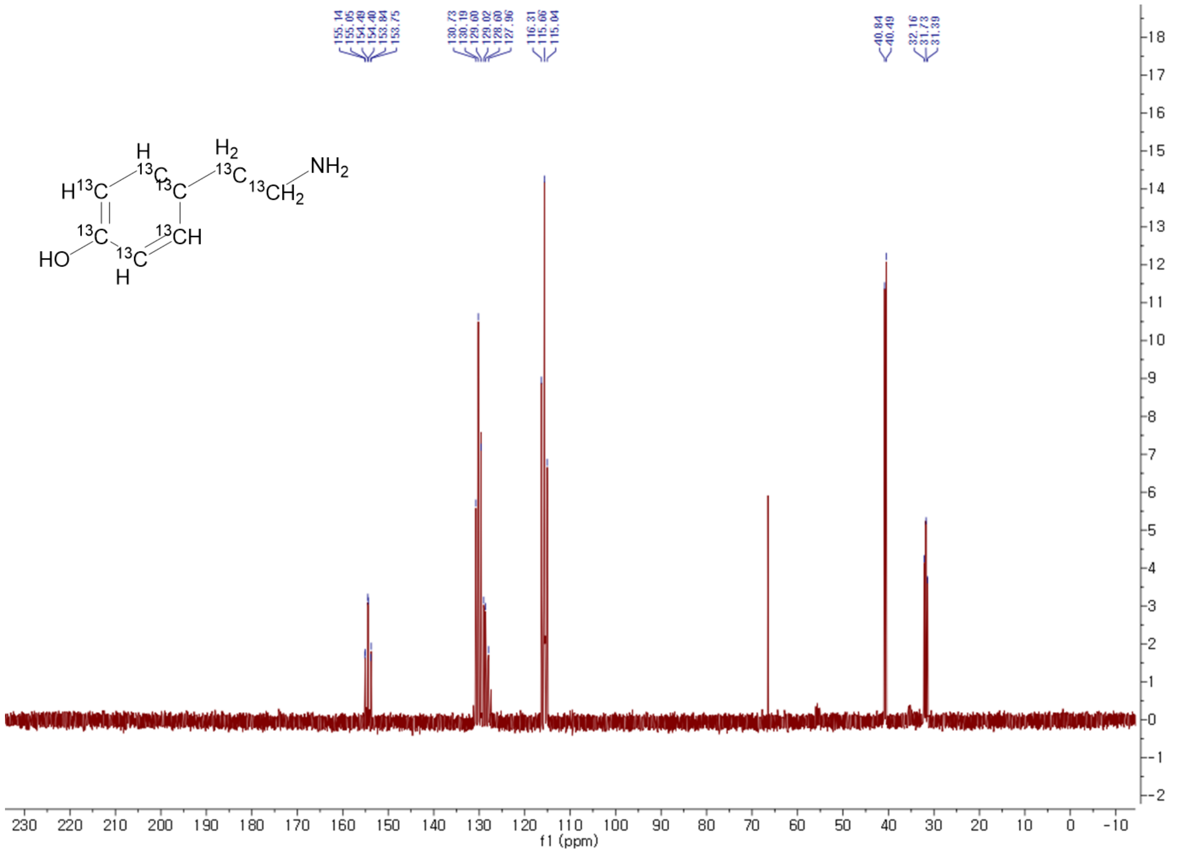

Supplementary Figure 18. (**a-b**) ^{1}H-NMR (**a**) and ^{13}C-NMR (**b**) of [$^{13}C_8$] tyramine in D_2O.

a

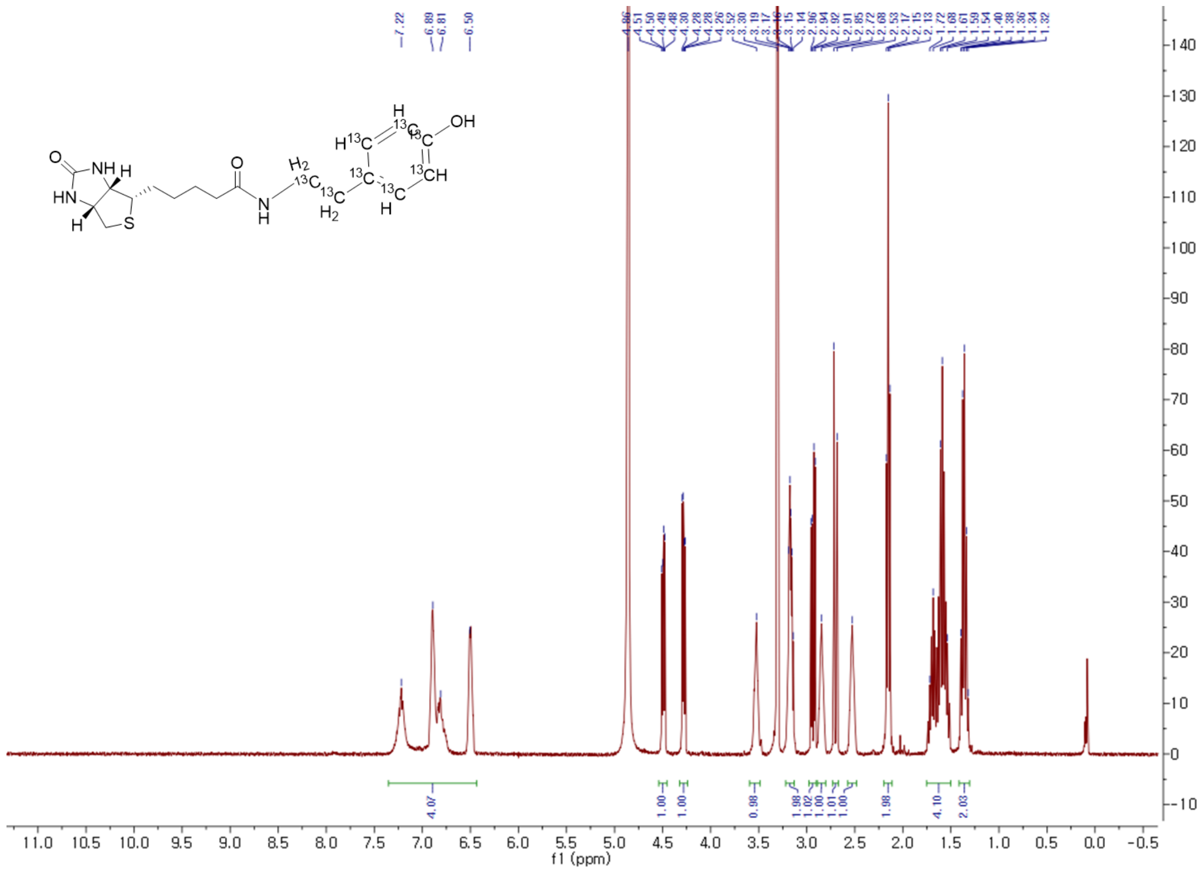

b

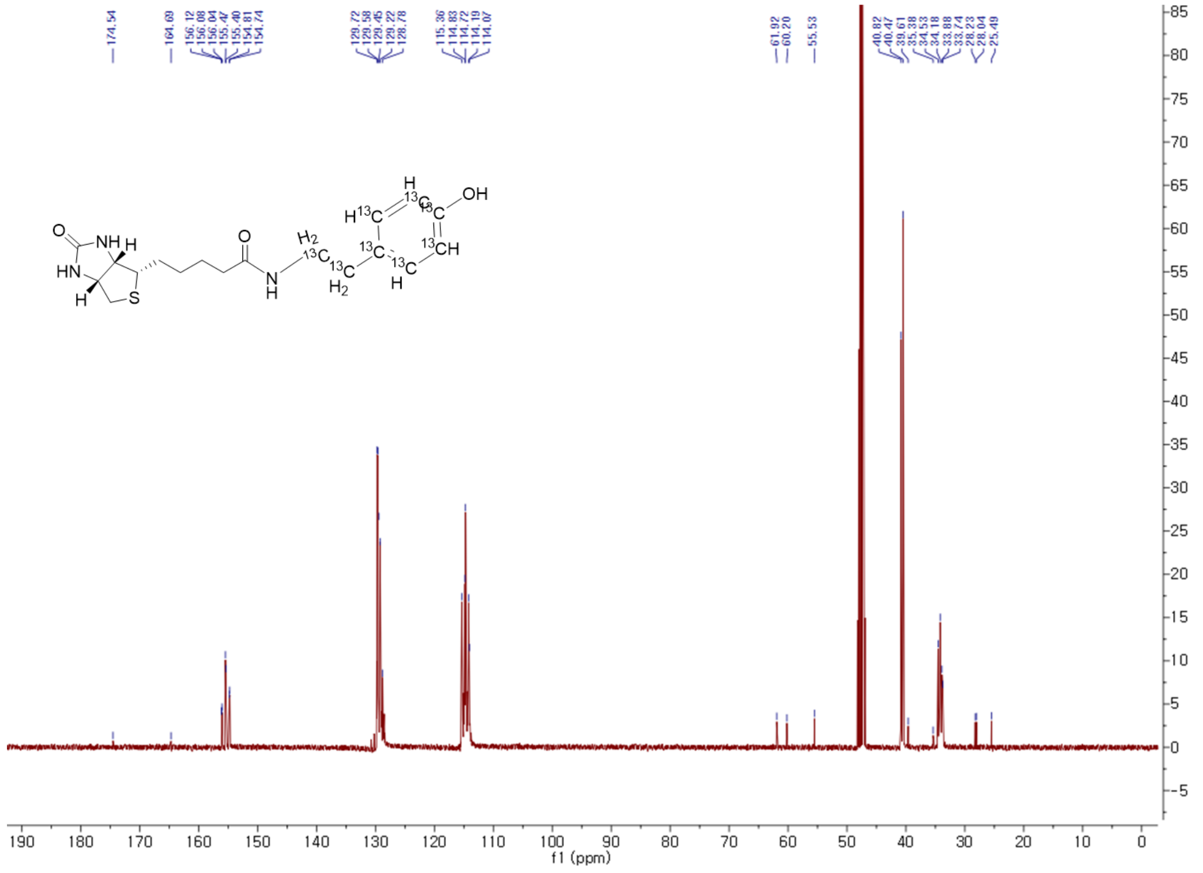

Supplementary Figure 19. (**a-b**) ^{1}H-NMR (**a**) and ^{13}C-NMR (**b**) of d-biotinyl[$^{13}C_8$] tyramine (HBP) in CD_3OD.

a

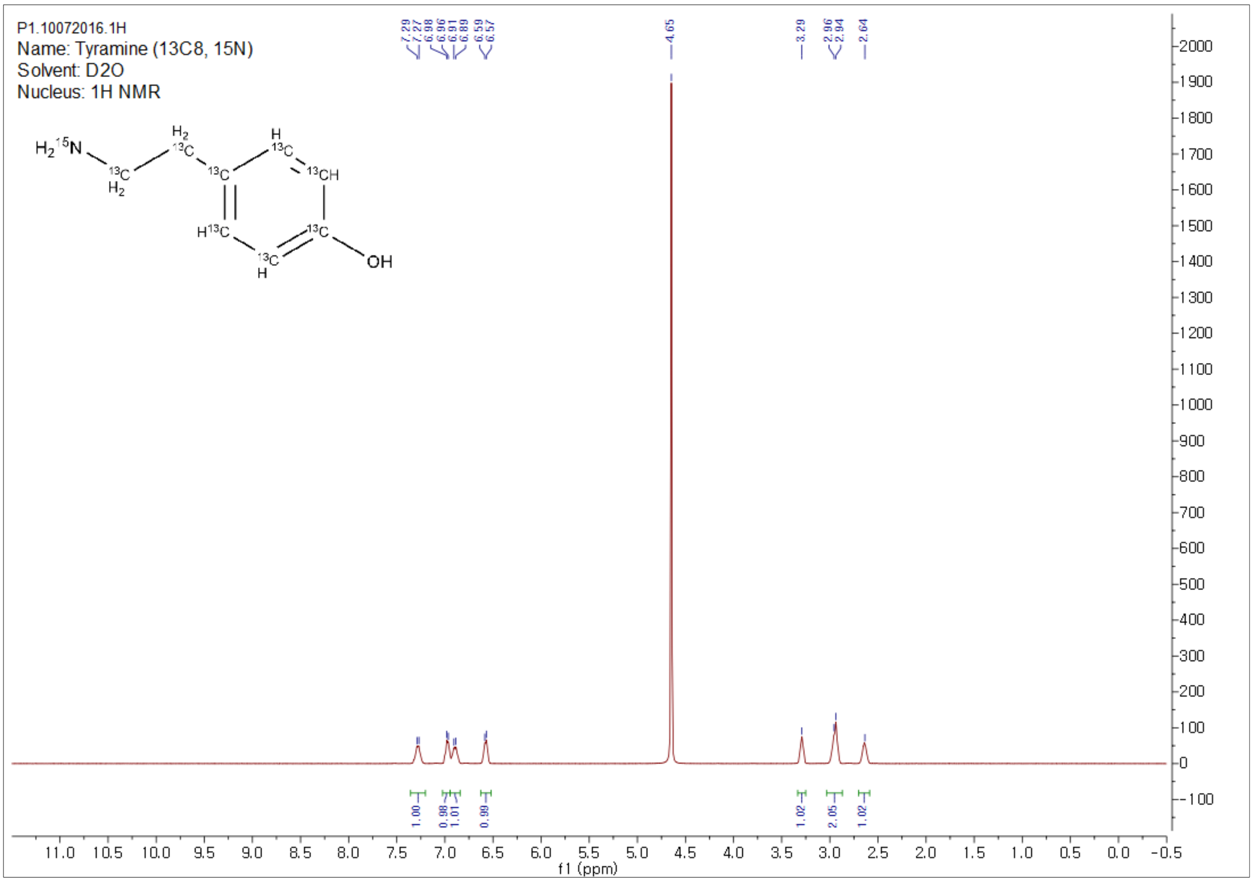

b

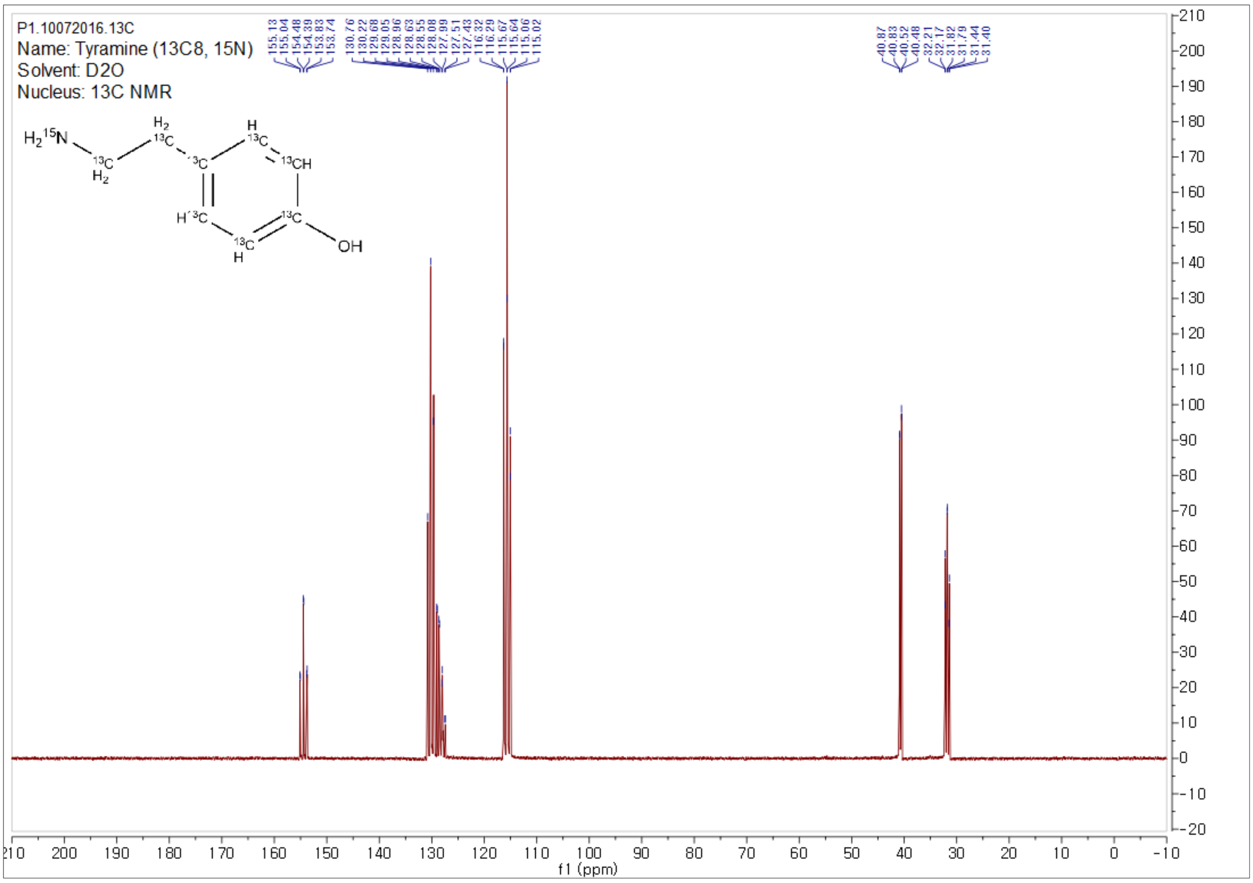

Supplementary Figure 20. (**a-b**) ^{1}H-NMR (**a**) and ^{13}C-NMR (**b**) of [$^{13}C_8$, ^{15}N] tyramine in D_2O.

a

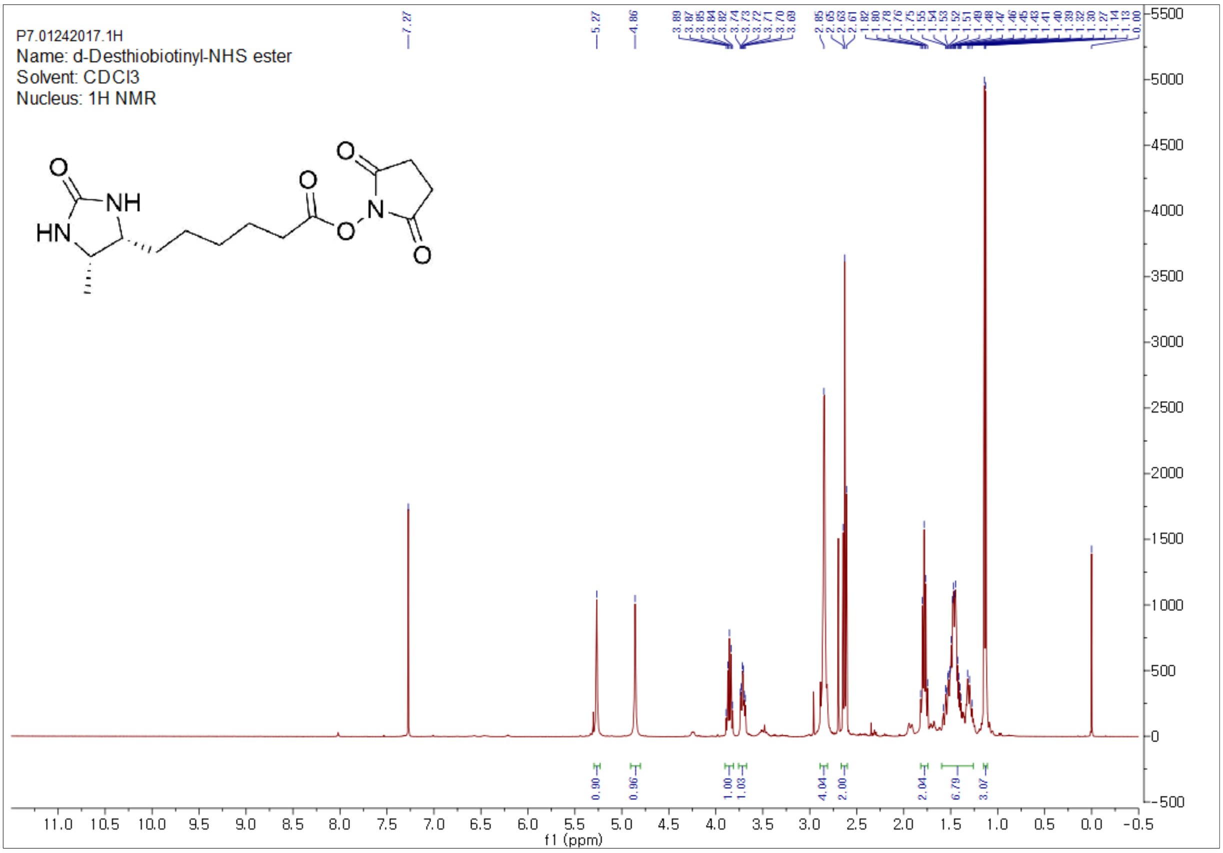

b

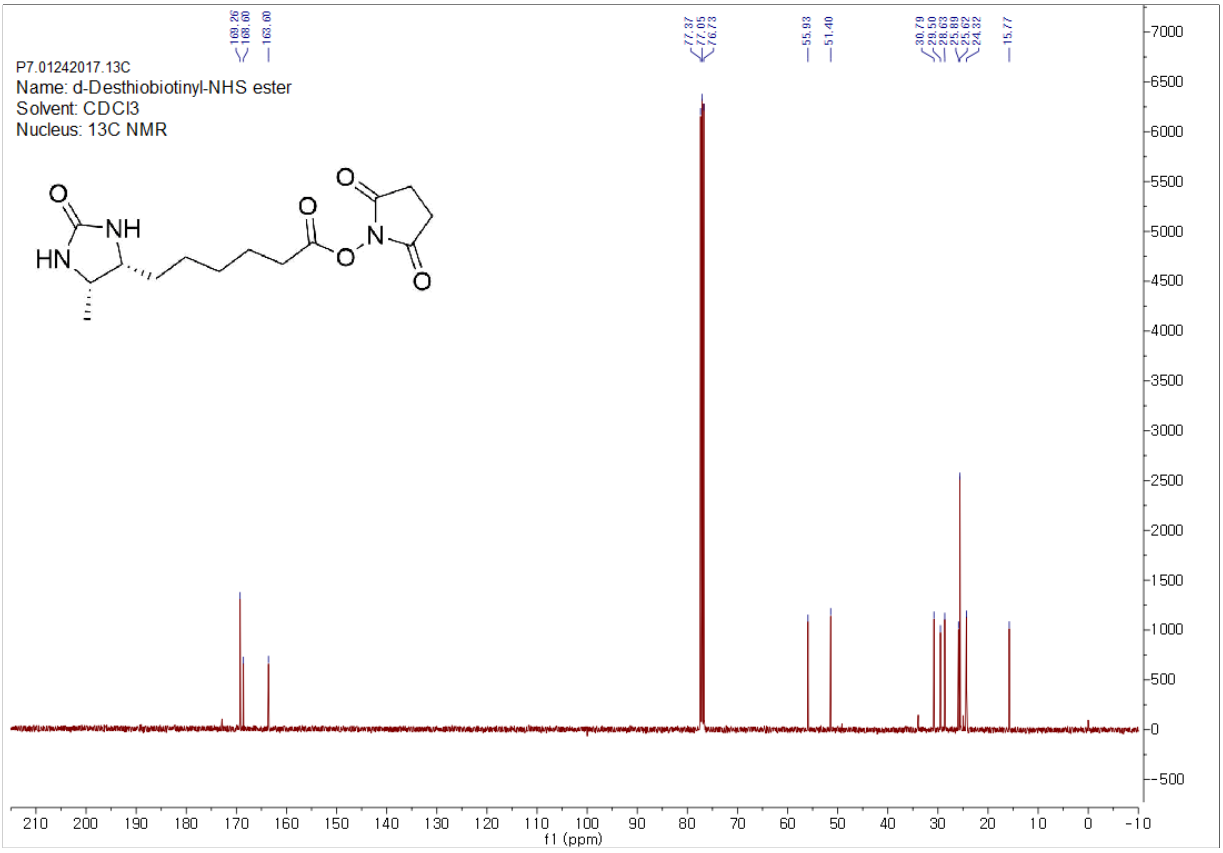

Supplementary Figure 21. (**a-b**) ^{1}H-NMR (**a**) and ^{13}C-NMR (**b**) of d-desthiobiotinyl-NHS ester in $CDCl_3$.

a

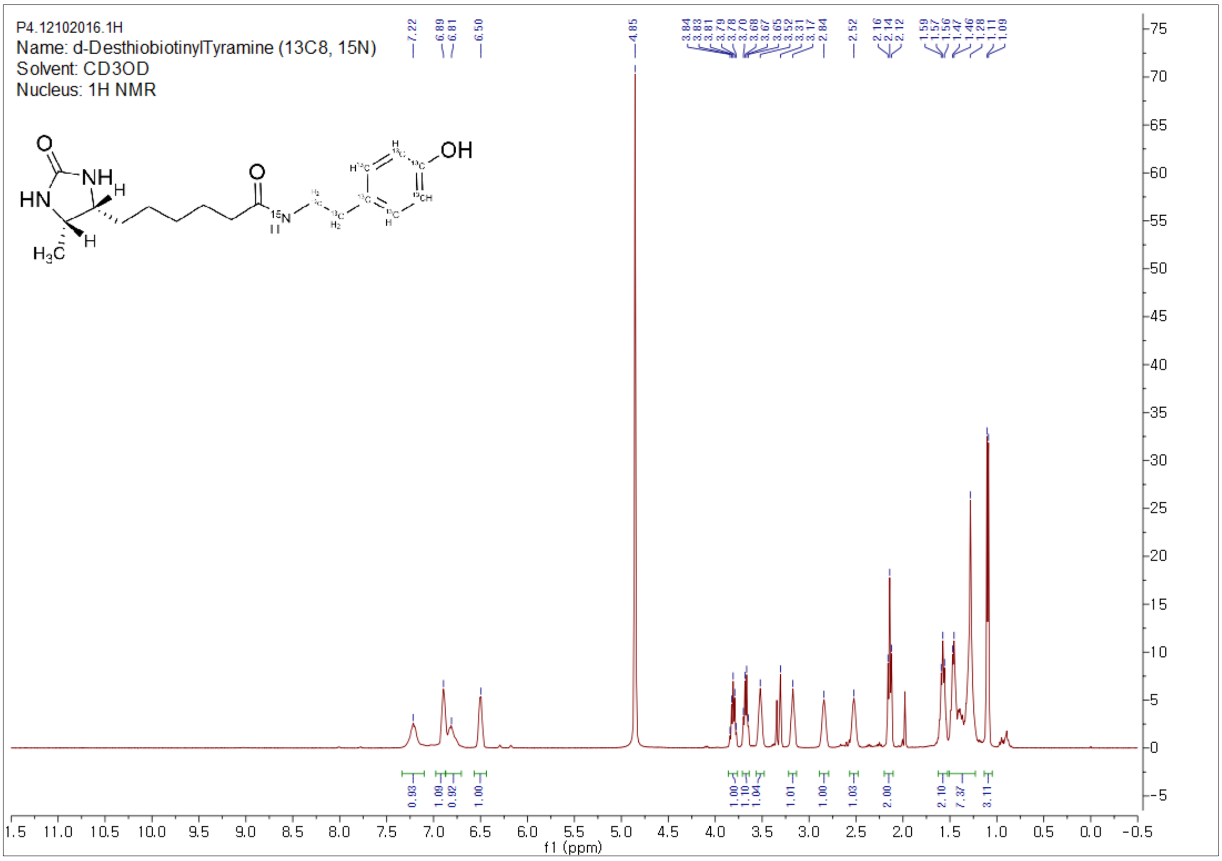

b

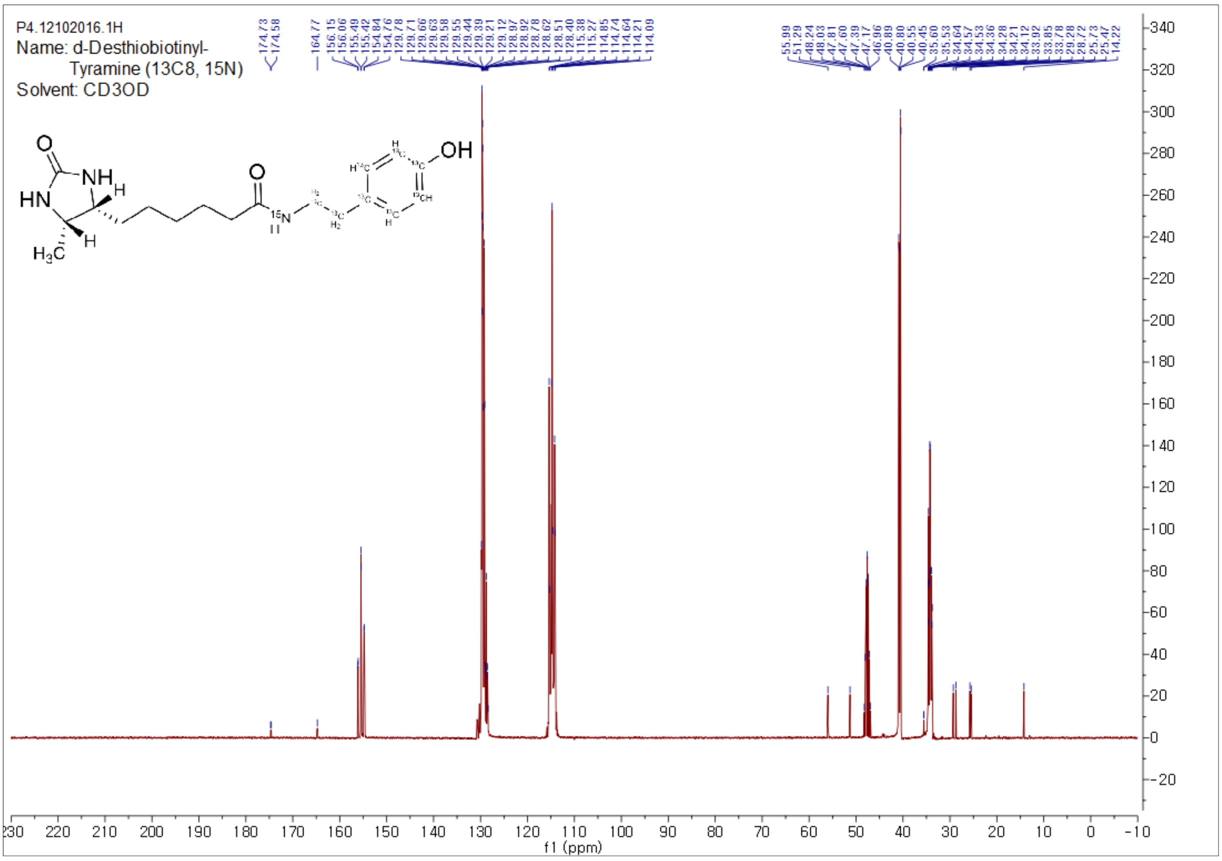

Supplementary Figure 22. (**a-b**) ^{1}H-NMR (**a**) and ^{13}C-NMR (**b**) of d-desthiobiotinyl[$^{13}C_8$, ^{15}N] tyramine (HDBP) in CD_3OD.

a

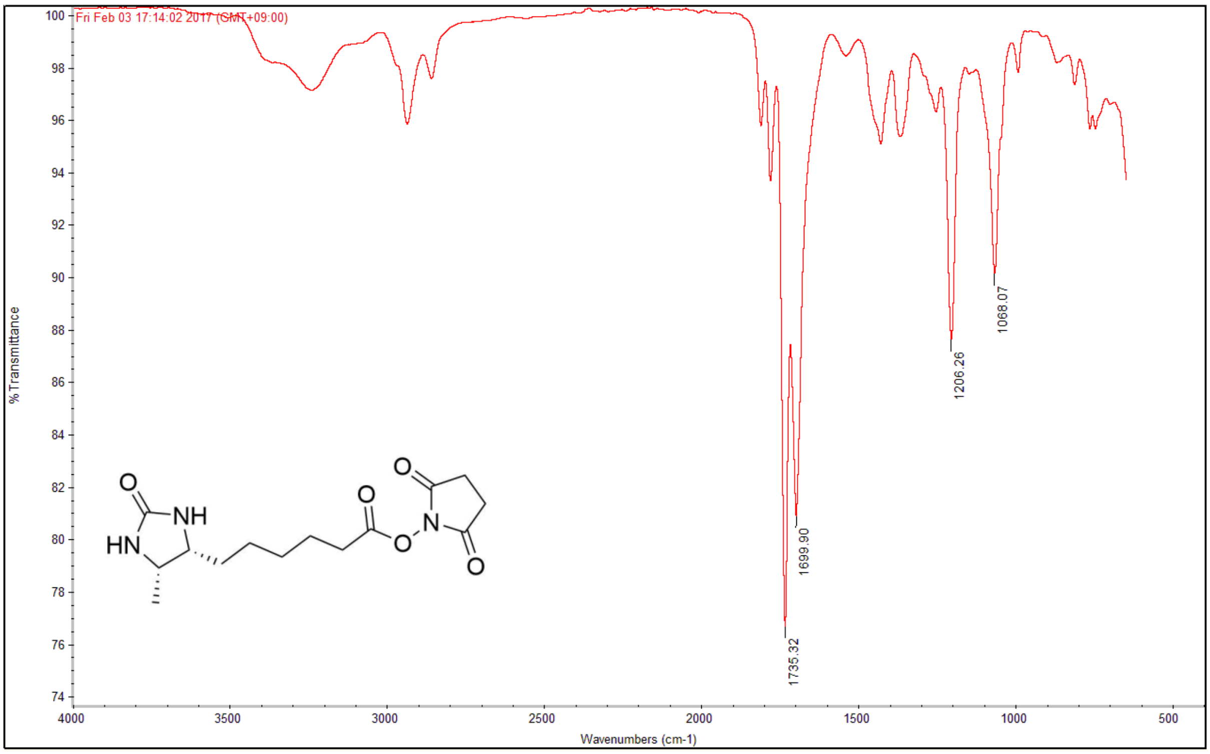

b

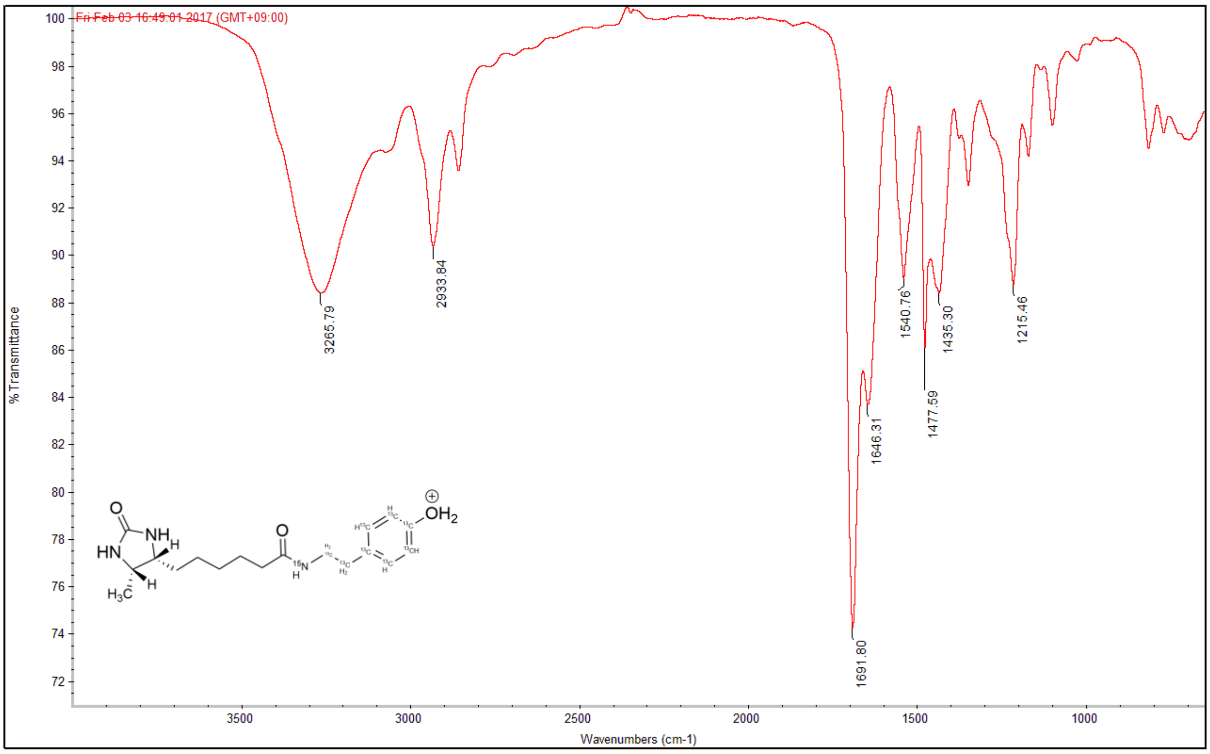

Supplementary Figure 23. (**a, b**) Infrared (IR) spectra of d-desthiobiotinyl-NHS ester (**a**) and d-desthiobiotinyl[$^{13}C_8$, ^{15}N] tyramine (**b**).

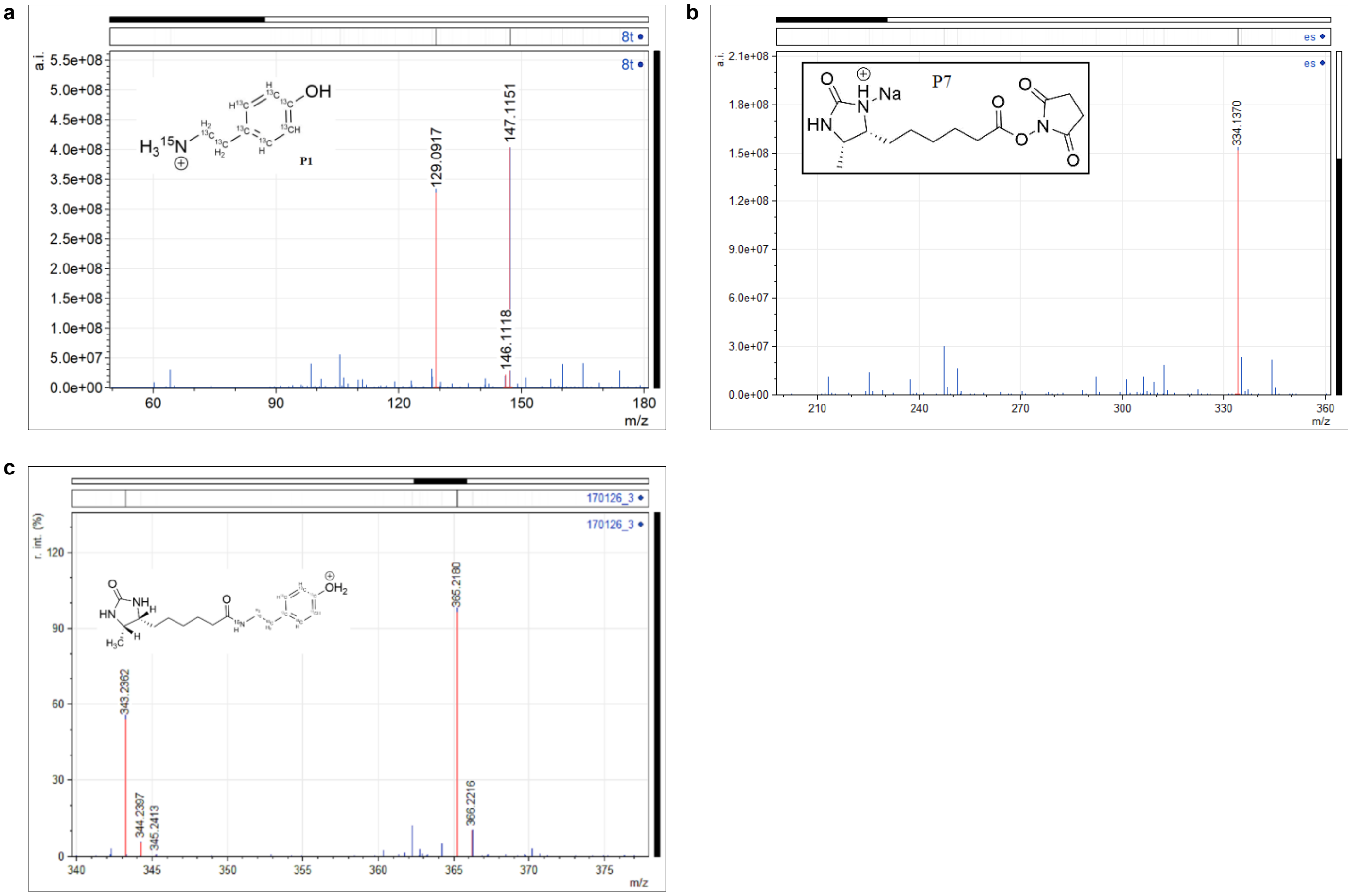

Supplementary Figure 24. (**a-c**) High-resolution mass spectrum of [$^{13}C_8$, ^{15}N] tyramine (**a**), d-desthiobiotinyl-NHS ester (**b**), and d-desthiobiotinyl[$^{13}C_8$, ^{15}N] tyramine (**c**).

Supplementary Methods

Biotin-phenol (Light biotin-phenol, LBP) and desthiobiotin-phenol (Light desthiobiotin-phenol, LDBP) were synthesized and characterized by reported procedure[1]. Heavy biotin-phenol (HBP) and heavy desthiobiotin-phenol (HDBP) were synthesized and characterized following the synthetic procedure.

Synthesis of [$^{13}C_8$]tyramine

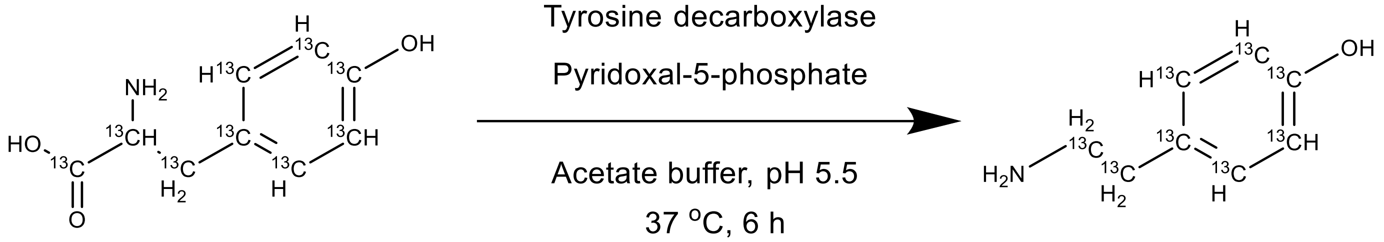

The protocol for decarboxylation of $^{13}C_9$-tyrosine generally followed the protocol described by Ntai *et al*[2]. with a few modifications. 100 mg of [$^{13}C_9$]tyrosine (Cambridge isotope laboratory, CLM-2263-H-PK) was dissolved in 100 ml H_2O by intermittent microwave heating until gentle boiling. The solution was cooled to room temperature, and a suspension of tyrosine decarboxylase apoenzyme from *Streptococcus faecalis* (2 mg, 0.05 unit/mg, from Sigma-Aldrich) and pyridoxal-5-phosphate (7 mg, Alfa aesar, A1232303) in sodium acetate buffer (4 ml, 0.1 M, pH 5.5) was added. The mixture was incubated at 37 °C for 6 hours with stirring. The reaction was stopped by brief heating to boiling, after which the reaction mixture was concentrated in vacuo and passed through a Dowex 50WX cation exchange column (hydrogen form). The tyramine product was eluted from the resin with 50 ml of 1 mM HCl and the solvent was evaporated to give [$^{13}C_8$]tyramine hydrochloride as a tan solid (88 mg, 93% yield).

TLC R_f = 0.5 (5% v/v NH_4OH solution in methanol)

^{1}H NMR (400 MHz, D_2O) δ 7.08 (d, J = 156.9 Hz, 2H), 6.77 (d, J = 164.4 Hz, 2H), 3.12 (d, J = 132.4 Hz, 2H), 2.78 (d, J = 120.0 Hz, 2H) ppm.

^{13}C NMR (100 MHz, D_2O) δ 154.45 (td, J = 65.4, 8.6 Hz, quaternary ^{13}C), 130.19 (t, J = 54.2 Hz, 2C, aromatic ^{13}C), 128.77 (m, quaternary ^{13}C), 115.66 (t, J = 62.0 Hz, quaternary ^{13}C), 40.67 (d, J = 34.9 Hz), 31.81 (t, J = 38.9 Hz) ppm.

MS (ESI) for $^{13}C_8H_{11}NO$ $[M+H]^+$ 146.12 (cald.), 146.583 (observed)

Synthesis of *d*-biotinyl[$^{13}C_8$]tyramine (HBP).

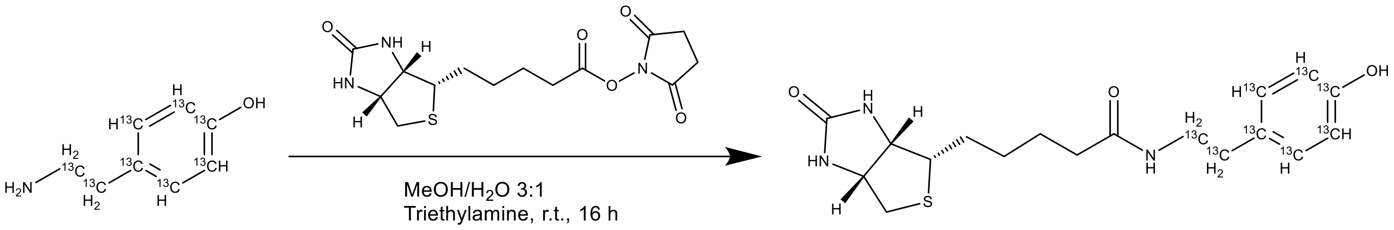

[$^{13}C_8$]tyramine hydrochloride (100 mg, 0.56 mmol) was dissolved in 5 ml of methanol/water (3:1, v/v). D-biotinyl-NHS ester (204 mg, 0.6 mmol) and triethylamine (83 µl, 0.6 mmol) were added to the tyramine solution. An additional 5 ml of methanol was added, and the resulting solution was stirred at room temperature for 16 hours. The reaction mixture was concentrated *in vacuo*, and the residue was diluted with water (50 ml) and extracted with ethyl acetate (50 ml x 8 times). The combined organic layers were dried over anhydrous magnesium sulfate, filtered, and concentrated *in vacuo*. The crude product was purified by flash column chromatography (ethyl acetate:methanol = 5:1 to yield a white powder (132 mg, 0.35 mmol, 63%).

TLC Rf = 0.3 (ethyl acetate/methanol = 5:1)

The purified product was analyzed using a reverse-phase analytical HPLC (Vydac C4 column, linear gradient of 0 to 73% eluent B over 30 min, 1 ml/min).

Eluent A = 0.1% TFA, Eluent B = 0.1% TFA in 90% acetonitrile/water (v/v)

^{1}H NMR (CD_3OD, 400 MHz) δ 7.02 (d, J = 158.5 Hz, 2H, aromatic H on tyramine), 6.70 (d, J=154.5 Hz, 2H, aromatic H on tyramine), 4.49 (dd, J = 7.9, 4.8 Hz, 1H, C**H$_a$**N), 4.28 (dd, J = 7.9, 4.5 Hz, C**H$_b$**N), 3.35 (d, J = 138.9 Hz, 2H, OCNH^{13}C**H$_2$**), 3.17 (m, 1H, SC**H**CH$_b$), 2.93 (dd, J = 12.8, 5.0 Hz, 1H, C$_H$**HS**) 2.70 (d, J = 12.7 Hz, 1H, C**H**$_H$S), 2.69 (d, J = 127.6 Hz, 2H, NHCH$_2$C**H$_2$**), 2.15 (t, J = 7.3 Hz, 2H, C**H$_2$**CO), 1.72-1.33(m, 6H, (C**H$_2$**)$_3$CH$_2$CO) ppm.

^{13}C NMR (CD_3OD, 100 MHz) δ 174.54 (**C**H$_2$CONH), 164.69 (NH**C**ONH), 155.47 (mt, quaternary ^{13}C), 129.59 (m, 2C, aromatic ^{13}C), 128.78 (m, quaternary ^{13}C), 114.72 (m, 2C, aromatic ^{13}C), 61.91 (CH$_b$N), 60.20 (**C**H$_a$N), 55.55 (**C**HS), 40.65 (d, J = 35.0 Hz, NH**^{13}C**H$_2$), 39.61(**C**H$_2$S), 35.38 (**C**H$_2$CO), 34.08 (mt, NH^{13}CH$_2$**^{13}C**H$_2$), 28.24 (**C**H$_2$), 28.05 (**C**H$_2$), 25.51(**C**H$_2$) ppm.

HRMS for $C_{10}{}^{13}C_8H_{25}N_3O_3S$ $[M+H]^+$ 372.1958 (cald.), 372.1950 (observed)

Synthesis of [$^{13}C_8$, ^{15}N]tyramine.

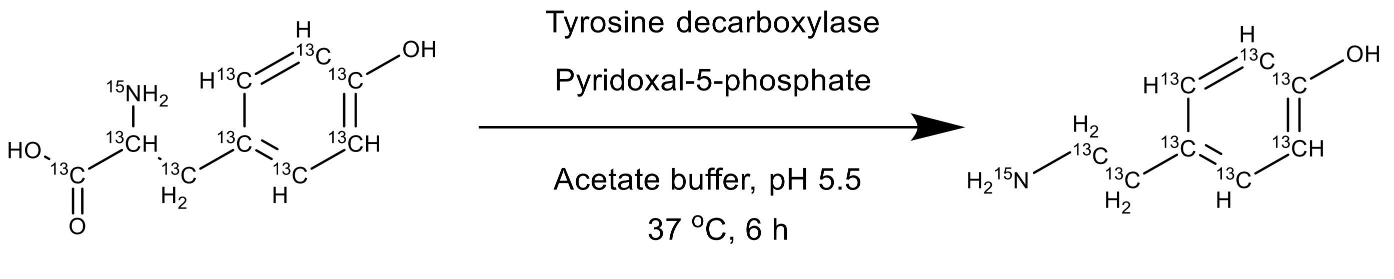

The protocol for decarboxylation of d_4-tyrosine as described by Ntai *et al*[2] was followed with a few modifications. [$^{13}C_9$, ^{15}N] tyrosine (100 mg) was dissolved in 100 ml H_2O by microwave heating until gentle boiling. The solution was cooled to room temperature, and a suspension of tyrosine decarboxylase from *Streptococcus faecalis* (2 mg, 0.05 unit/mg) and pyridoxal-5-phosphate (7 mg, Alfa aesar, A1232303) in sodium acetate buffer (4 ml, 0.1 M, pH 5.5) was added. The mixture was incubated at 37 °C for 6 hours with stirring. The reaction was stopped by brief heating to boiling, after which the reaction mixture was concentrated in vacuo and passed through a Dowex 50WX cation exchange column (hydrogen form). The tyramine product was eluted from the resin with approximately 50 ml of 1 mM HCl, and the solvent was evaporated to give [$^{13}C_8$, ^{15}N] tyramine as a tan solid (74 mg, 97% yield).

TLC R_f = 0.5 (5% v/v NH_4OH solution in methanol)

^{1}H NMR (400 MHz, D_2O) δ 7.09 (dd, *J* = 153.6, 8.4 Hz, ring 2**^{13}CH,** 2H), 6.77 (dd, *J* = 158.0, 6.3 Hz, ring 2**^{13}CH,** 2H), 3.12 (d, *J* = 134.0 Hz, **$^{13}CH_2$**$^{15}NH_2$, 2H), 2.79 (d, *J* = 120.9 Hz, ^{13}C**$^{13}CH_2$**, 2H) ppm.

^{13}C NMR (101 MHz, D_2O) δ 154.44 (td, *J* = 65.3, 8.7 Hz), 130.22 (t, *J* = 54.4 Hz), 128.40 (m), 115.67 (td, *J* = 64.0, 62.8, 3.6 Hz), 40.68 (dd, *J* = 35.0, 4.0 Hz), 31.81 (td, *J* = 39.3, 4.4 Hz) ppm.

HRMS (ESI) Calcd. for $^{13}C_8H_{12}{}^{15}NO^+$; 147.1152 m/z $(M+H)^+$, Observed; 147.1151 m/z $(M+H)^+$

Synthesis of d-desthiobiotinyl-NHS ester.

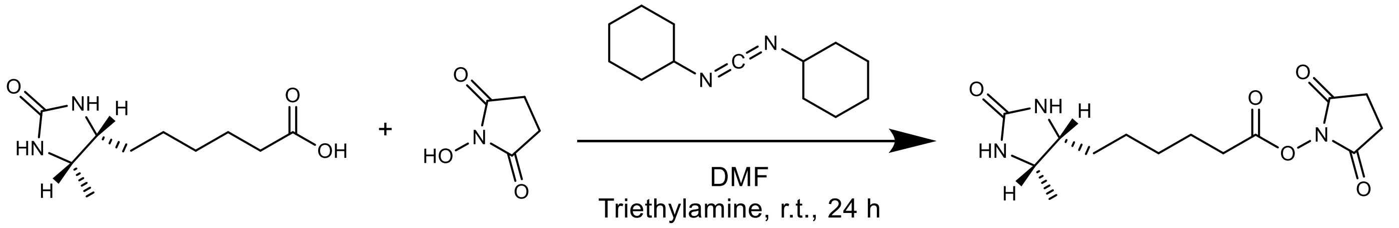

D-desthiobiotin (1.3 g, 6.07 mmol) was dissolved in 20 mL of DMF, and N, N'-dicyclohexylcarbodiimide (DCC, 1.37 g, 6.67 mmol), N-hydroxysuccinimide (NHS; 0.91 g, 7.89 mmol) and triethylamine (1.3 mL, 9.10 mmol) were added. The mixture was stirred at room temperature for 24 h. The reaction mixture was filtered to remove precipitated solids and concentrated *in vacuo*. The resulting solid was dissolved in saturated aqueous ammonium chloride solution and extracted with ethyl acetate. The combined organic layers were evaporated to dryness, yielding the product as a white powder. The crude product was used in the next step without further purification (1.6 g, 5.14 mmol, 85 % yield).

TLC Rf = 0.1 (ethyl acetate/MeOH = 4:1)

^{1}H NMR (400 MHz, $CDCl_3$) δ 5.27 (s, CO**NH**, 1H), 4.86 (s, CO**NH**, 1H), 3.86 (p, J = 6.6 Hz, **CH**CH_3, 1H), 3.71 (td, J = 8.1, 3.9 Hz, **CH**CH_2, 1H), 2.85 (s, 2NCO**CH_2**, 4H), 2.63 (t, J = 7.2 Hz, **CH_2**COO, 2H), 1.78 (p, J = 7.3 Hz, CH**CH_2**, 1H), 1.58-1.27 (m, 6H), 1.13 (d, J = 6.5 Hz, CH**CH_3**, 3H) ppm.

^{13}C NMR (101 MHz, $CDCl_3$) δ 127.87, 169.26, 168.60, 163.60, 55.93, 51.40, 29.50, 28.63, 25.89, 25.62, 24.32, 15.77 ppm.

HRMS (ESI) Calcd for $C_{14}H_{21}N_3O_5Na^+$; 334.1373 m/z $(M+Na)^+$ Observed; 334.1370 m/z $(M+Na)^+$.
IR 1735.32, 1699.90, 1206.26, 1068.07 cm^{-1}

Synthesis of d-desthiobiotinyl[$^{13}C_8$, ^{15}N]tyramine (HDBP).

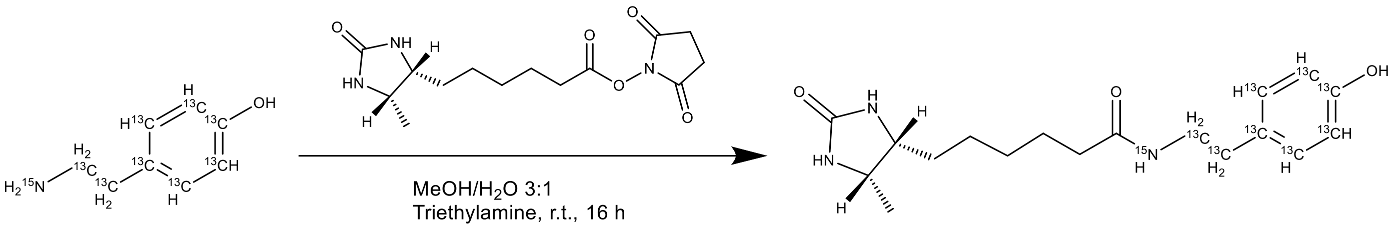

[$^{13}C_8$, ^{15}N] tyramine (190.8 mg, 1.32 mmol) was dissolved in 5 ml of methanol/water (3:1, v/v). D-desthiobiotinyl-NHS ester (450.1 mg, 1.44 mmol) and triethylamine (209 μl, 1.44 mmol) were added to the tyramine solution. An additional 5 ml of methanol was added, and the resulting solution was stirred at room temperature for 16 hours. The reaction mixture was concentrated *in vacuo* and the residue was diluted with water (50 ml) and extracted with ethyl acetate (50 ml x 8 times). The combined organic layers were dried over anhydrous magnesium sulfate, filtered, and concentrated *in vacuo*. The crude product was purified by reverse-phase HPLC (C_{18} column, 10 ml/min, linear gradient of 15 to 35% acetonitrile/water over 40 min, retention time 10 min). The purified product was lyophilized to yield white powder (250 mg, 0.75 mmol, 57%).
TLC R_f = 0.85 (ethyl acetate/methanol = 3:1)
The purified product was analyzed using a reverse-phase analytical HPLC Analytical (method: 20-40% solvent B over 15 min. TR = 12.043 min).

Solvent A: deionized water with 0.1% TFA

Solvent B: 9:1 of MeCN and water with 0.1% TFA

^{1}H NMR (400 MHz, CD_3OD) δ 7.02 (dd, *J* = 154.3, 6.7 Hz, ring 2^{13}**CH**, 2H), 6.70 (d, *J* = 158.6 Hz, ring 2^{13}**CH**, 2H), 3.81 (p, *J* = 6.5 Hz, **CH**CH_3, 1H), 3.67 (q, *J* = 6.9 Hz, **CH**CH_2CH_2, 1H), 3.35 (d, *J* = 138.8 Hz, ^{15}NH^{13}**CH₂**, 2H), 2.68 (d, *J* = 126.6 Hz, C^{13}**CH₂**, 2H), 2.14 (t, *J* = 7.3 Hz, **CH₂**CONH, 2H), 1.57 (t, *J* = 7.0 Hz, CH**CH₂**, 2H), 1.49 – 1.28 (m, CCH_2**CH₂CH₂CH₂**, 6H), 1.10 (d, J = 6.4 Hz, CH**CH₃**, 3H) ppm.
^{13}C NMR (101 MHz, CD_3OD) δ 174.65 (d, *J* = 14.9 Hz), 164.77, 155.45 (td, *J* = 66.6, 8.3 Hz), 129.56 (ddd, *J* = 18.6, 12.2, 5.3Hz), 129.02 (m), 114.74 (ddd, *J* = 65.5 54.6, 11.8 Hz), 55.99, 51.29, 40.67 (dd, *J* = 35.0, 10.0 Hz), 35.57 (d, *J* = 7.1 Hz), 34.23 (ddt, *J* = 44.1 35.1, 7.5 Hz), 29.28, 28.72, 25.78, 25.47, 14.22 ppm.
HRMS (ESI) Calcd. for $C_{10}{}^{13}C_8H_{27}N_2{}^{15}NO_3$; 343.2364 m/z $(M+H)^+$, 365.2183 m/z $(M+Na)^+$ Observed; 343.2362 m/z $(M+H)^+$ 365.2180 m/z $(M+Na)^+$
IR 3265.79, 2933.84, 1691.80, 1643.31, 1540.76, 1477.59, 1435.30, 1215.46 cm^{-1}

Supplementary References

1. Lee, S.Y., Kang, M.G., Shin, S., Kwak, C., Kwon, T., Seo, J.K., Kim, J.S., and Rhee, H.W. (2017). Architecture Mapping of the Inner Mitochondrial Membrane Proteome by Chemical Tools in Live Cells. J Am Chem Soc *139*, 3651-3662. 10.1021/jacs.6b10418.
2. Ntai, I., Phelan, V.V., and Bachmann, B.O. (2006). Phosphonopeptide K-26 biosynthetic intermediates in Astrosporangium hypotensionis. Chem Commun (Camb), 4518-4520. 10.1039/b611768f.
