# Supplementary material for: Intracristal space proteome mapping using super-resolution proximity labeling with isotope-coded probes
Source: Nat Commun. 2025 Aug 20;16:7757. doi: 10.1038/s41467-025-62756-0 (PMC12368266; doi:10.1038/s41467-025-62756-0)
Supplement: Supplementary file 2 — Description of Additional Supplementary Files [file 41467_2025_62756_MOESM2_ESM.pdf]

Description of Additional Supplementary Files

File name: Supplementary Data 1
Description: TurboID-NES labeling under endogenous and excess biotin conditions.

File name: Supplementary Data 2
Description: Validation of isotopic purity of LDBP and HDBP probes using stable cells expressing MTS-APEX2.

File name: Supplementary Data 3
Description: Equal activity test of LDBP and HDBP using stable cells expressing MTS-APEX2. LDBP and HDBP labeling in the MTS-APEX2 cells for ICAX analysis.

File name: Supplementary Data 4
Description: Label-free quantification of either LDBP- or HDBP-modified proteins labeled by MTS-APEX2.

File name: Supplementary Data 5
Description: Activity test of biotin-conjugated probes in stable cells expressing MTS-APEX2 following treatment of equal molar concentration of mixed probes. ICAX analysis with LBP and HBP using MTS-APEX2 cells.

File name: Supplementary Data 6
Description: Identification of ICS proteins using cells stably expressing SCO1-APEX2 and TDRKH-APEX2 with LDBP and HDBP.

File name: Supplementary Data 7
Description: Mapping of the ICS and OCS proteomes using cells stably expressing TMEM177-APEX2 and AGK-APEX2 with LDBP and HDBP.

File name: Supplementary Data 8
Description: Identification of changed proteome in aberrant ICS induced by MICOS complex inhibition using TMEM177-APEX2 with LDBP and HDBP.

File name: Supplementary Data 9
Description: Monitoring of mitochondrial dynamics using cells stably expressing TMEM177-APEX2 under mitochondrial uncoupling conditions with LDBP and HDBP.

File name: Supplementary Data 10
Description: Optimization of TMT labeling and isobaric quantification of DBP-modified peptides labeled by MTS-APEX2 using LDBP.

File name: Supplementary Data 11
Description: Detailed information of the plasmids used in this study.
