# Supplementary material for: Intracristal space proteome mapping using super-resolution proximity labeling with isotope-coded probes
Source: Nat Commun. 2025 Aug 20;16:7757. doi: 10.1038/s41467-025-62756-0 (PMC12368266; doi:10.1038/s41467-025-62756-0)
Supplement: Supplementary file 14 — Reporting Summary [file 41467_2025_62756_MOESM14_ESM.pdf]

nature portfolio

Corresponding author(s): Hyun-Woo Rhee

Last updated by author(s): Jul 12, 2025

Reporting Summary

Nature Portfolio wishes to improve the reproducibility of the work that we publish. This form provides structure for consistency and transparency in reporting. For further information on Nature Portfolio policies, see our Editorial Policies and the Editorial Policy Checklist.

Statistics

For all statistical analyses, confirm that the following items are present in the figure legend, table legend, main text, or Methods section.

n/a	Confirmed	
☐	☒	The exact sample size (*n*) for each experimental group/condition, given as a discrete number and unit of measurement
☐	☒	A statement on whether measurements were taken from distinct samples or whether the same sample was measured repeatedly
☐	☒	The statistical test(s) used AND whether they are one- or two-sided<br>*Only common tests should be described solely by name; describe more complex techniques in the Methods section.*
☒	☐	A description of all covariates tested
☒	☐	A description of any assumptions or corrections, such as tests of normality and adjustment for multiple comparisons
☐	☒	A full description of the statistical parameters including central tendency (e.g. means) or other basic estimates (e.g. regression coefficient) AND variation (e.g. standard deviation) or associated estimates of uncertainty (e.g. confidence intervals)
☐	☒	For null hypothesis testing, the test statistic (e.g. *F*, *t*, *r*) with confidence intervals, effect sizes, degrees of freedom and *P* value noted<br>*Give P values as exact values whenever suitable.*
☒	☐	For Bayesian analysis, information on the choice of priors and Markov chain Monte Carlo settings
☒	☐	For hierarchical and complex designs, identification of the appropriate level for tests and full reporting of outcomes
☒	☐	Estimates of effect sizes (e.g. Cohen's *d*, Pearson's *r*), indicating how they were calculated

Our web collection on statistics for biologists contains articles on many of the points above.

Software and code

Policy information about availability of computer code

Data collection	LC-MS/MS data generated in this study were searched by MaxQuant (version 1.6.2.3) with Andromeda search engine at 10 ppm precursor ion mass tolerance against the SwissProt Homo sapiens proteome database (20,199 entries, UniProt (http://www.uniprot.org/).
Data analysis	The data for TMT labeling was searched by Proteome Discoverer (version 2.4) with SEQUEST search engine at 10 ppm precursor ion mass tolerance against the SwissProt Homo sapiens proteome database. Statistical tests and informatic analysis of LC-MS/MS data were performed using Perseus (v.1.6.2.3) with implemented WGCNA R-package, InfernoRDN, R studio, and Microsoft excel. Unpaired two-tailed t-test was performed using a GraphPad prism (v.10.4.2). Seahorse XF Wave software v2.6 (Agilent) was used for displaying and exporting the data of oxygen consumption rate (OCR) measurement.

For manuscripts utilizing custom algorithms or software that are central to the research but not yet described in published literature, software must be made available to editors and reviewers. We strongly encourage code deposition in a community repository (e.g. GitHub). See the Nature Portfolio guidelines for submitting code & software for further information.

Data

Policy information about availability of data

All manuscripts must include a data availability statement. This statement should provide the following information, where applicable:

- Accession codes, unique identifiers, or web links for publicly available datasets
- A description of any restrictions on data availability
- For clinical datasets or third party data, please ensure that the statement adheres to our policy

The proteomics data generated in this study has been deposited in the ProteomeXchange database under accession code PXD039492 [https://proteomecentral.proteomexchange.org/cgi/GetDataset?ID=PXD039492].

Research involving human participants, their data, or biological material

Policy information about studies with human participants or human data. See also policy information about sex, gender (identity/presentation), and sexual orientation and race, ethnicity and racism.

Reporting on sex and gender	Not applicable (N/A) in this study.
Reporting on race, ethnicity, or other socially relevant groupings	N/A
Population characteristics	N/A
Recruitment	N/A
Ethics oversight	N/A

Note that full information on the approval of the study protocol must also be provided in the manuscript.

Field-specific reporting

Please select the one below that is the best fit for your research. If you are not sure, read the appropriate sections before making your selection.

☒ Life sciences ☐ Behavioural & social sciences ☐ Ecological, evolutionary & environmental sciences

For a reference copy of the document with all sections, see nature.com/documents/nr-reporting-summary-flat.pdf

Life sciences study design

All studies must disclose on these points even when the disclosure is negative.

Sample size	No statistical calculation was used to predetermine sample size. In the case of LC-MS/MS experiments, sample sizes were selected based on the previous study (for example, PMID: 32414919). For other experiments, sample sizes commonly used in this field were adopted. Relevant references for each experiment have been cited accordingly in the manuscript.
Data exclusions	Decoy and unmodified peptides, which were not modified by DBP or BP, were excluded from the proteomics data.
Replication	All experiments, including those involving MS data, were conducted with at least two independent biological replicates. The exact number of replicates is indicated in the corresponding figure legends or in the Methods section. The LC-MS/MS dataset showed high reproducibility in relative intensity among the replicates.
Randomization	Cells were equally seeded on the cell culture plates, but chemical (e.g., BAM15) was treated randomly.
Blinding	LC-MS/MS sample preparation and analysis were assigned to different groups. No detailed description was labeled on the sample tubes for blinding, and samples were only distinguished by numbers during injection onto mass spectrometer and analysis of data.

Reporting for specific materials, systems and methods

We require information from authors about some types of materials, experimental systems and methods used in many studies. Here, indicate whether each material, system or method listed is relevant to your study. If you are not sure if a list item applies to your research, read the appropriate section before selecting a response.

Materials & experimental systems

n/a	Involved in the study
☐	☒ Antibodies
☐	☒ Eukaryotic cell lines
☒	☐ Palaeontology and archaeology
☒	☐ Animals and other organisms
☒	☐ Clinical data
☒	☐ Dual use research of concern
☒	☐ Plants

Methods

n/a	Involved in the study
☒	☐ ChIP-seq
☒	☐ Flow cytometry
☒	☐ MRI-based neuroimaging

Antibodies

Antibodies used

Antibodies used in this study are listed as follow:
- Anti-V5 Tag Monoclonal Antibody (mouse) (Thermo Fisher Scientific, R960-25)
- Streptavidin, Horseradish Peroxidase Conjugate (Thermo Fisher Scientific, 21124)
- Streptavidin, Alexa Fluor™ 568 conjugate (Thermo Fisher Scientific, S11226)
- Streptavidin, Alexa Fluor™ 647 conjugate (Thermo Fisher Scientific, S21374)
- HRP-conjugated goat anti-rabbit IgG (H+L) (Cell Signaling Technology, 7074S)
- HRP-conjugated goat anti-mouse IgG (H+L) (Bio-Rad Laboratories, 1706516)
- Goat anti-Rabbit IgG (H+L) antibody, Alexa Fluor™ 408 (Thermo Fisher Scientific, A31556)
- Goat anti-Mouse IgG (H+L) antibody, Alexa Fluor™ 488 (Thermo Fisher Scientific, A11001)
- Goat anti-Rabbit IgG (H+L) antibody, Alexa Fluor™ 568 (Thermo Fisher Scientific, A11011)
- Goat anti-Rabbit IgG (H+L) antibody, Alexa Fluor™ 647 (Thermo Fisher Scientific, A21244)
- Anti-HSPD1 rabbit (Cell Signaling Technology, 12165)
- Anti-TOMM20 rabbit (ProteinTech, 11802-1-AP)
- Anti-CHCHD3 rabbit (Sigma-Aldrich, HPA042935)
- Anti-GAPDH mouse (Santa Cruz Biotechnology, sc-32233)
- Anti-beta actin mouse (Santa Cruz Biotechnology, sc-47778)
- Anti-Myc mouse (Santa Cruz Biotechnology, sc-40)
- Anti-GFP mouse (Thermo Fisher Scientific, MA5-15256)
- Anti-MIC60 mouse (Abcam, ab110329)

Validation

All antibodies are commercially available and validated by the supplier. In this study, we also confirmed the specificity of listed antibodies.

Eukaryotic cell lines

Policy information about cell lines and Sex and Gender in Research

Cell line source(s)

HEK293T cell line was obtained from the American Type Culture Collection (ATCC) and Flp-In™ T-REx™ 293 cell line was purchased from the Thermo Fisher Scientific. HeLa cell line was obtained from Korean Cell Line Bank.

Authentication

HEK293T and Flp-In™ T-REx™ 293 cell line were certified by ATCC and Thermo Fisher Scientific, respectively. HeLa cell line was certified by Korean Cell Line Bank.

Mycoplasma contamination

All cell lines were varified to be mycoplasma-negative by supplier.

Commonly misidentified lines
(See ICLAC register)

No misidentified cell line was used in this study.

Plants

Seed stocks

N/A

Novel plant genotypes

N/A

Authentication

N/A
